# External morphology and developmental changes of tarsal tips and mouthparts of the invasive spotted lanternfly, *Lycorma delicatula* (Hemiptera: Fulgoridae)

**DOI:** 10.1371/journal.pone.0226995

**Published:** 2019-12-26

**Authors:** Alina Avanesyan, Timothy K. Maugel, William O. Lamp

**Affiliations:** 1 Department of Entomology, University of Maryland, College Park, Maryland, United States of America; 2 Laboratory for Biological Ultrastructure, University of Maryland, College Park, Maryland, United States of America; Laboratoire de Biologie du Développement de Villefranche-sur-Mer, FRANCE

## Abstract

External structures of insects contribute to the ability of herbivores to select and feed on their host plants. The invasive spotted lanternfly, *Lycorma delicatula* (Hemiptera: Fulgoridae) is an economically important and polyphagous insect pest in the eastern US. The lanternfly causes substantial damage to many woody plants by sucking phloem sap, reducing photosynthesis, causing weeping wounds, and creating conditions for sooty mold. Lanternfly nymphs switch host plants during their development. However, little is known about relationship between the lanternfly and its plant hosts, and particularly about morphological adaptations of the lanternfly to host plant usage at each developmental stage of the pest. In this study, we focused on assessing changes in morphology of (a) the lanternfly mouthparts (stylets and labium), and (b) the lanternfly tarsal tips (arolia and tarsal claws) at each developmental stage. Our study revealed several developmental patterns among which the presence of the indentations on mandibular stylets in late instars and adults, as well as the exponential growth of the labium and stylet length, and the tarsal claw dispersal during the lanternfly development. Our findings are critical for investigating and predicting the lanternfly host range, and the lanternfly dispersal to new host trees at each developmental stage.

## Introduction

In natural ecosystems, insect herbivores display a wide range of adaptations to their host plants, as well as diverse feeding behavior. On the one hand, such diversity in feeding habits reflects the diversity in insect herbivore diet, i.e. the diversity in their host plants (e.g. plant chemistry, plant mechanical traits, etc.) [1]. On the other hand, insect herbivore diversity has a strong impact on plant diversity, primary production, and it influences other ecosystem processes, such as nutrient cycling [2]. Particularly, form and function of insect herbivore mouthparts, as well as insect ability to attach to their hosts, create the potential for consuming plant tissue, as well as provide a line of defense for host plants to protect themselves. In addition, host plant use may change with insect development, which might be potentially associated with variation in insect morphology at each developmental stage. In this study we focused on the emerging invasive insect herbivore, the spotted lanternfly, and described the external morphology of its mouthparts and tarsi at electron microscopical level and in relation to the lanternfly extensive host usage.

The spotted lanternfly, *Lycorma delicatula* (Hemiptera: Fulgoridae), is a recently introduced highly invasive insect pest in the US which poses a significant risk to forestry and agriculture [3,4,5,6]. It originated from and is widely distributed in China, but due to its highly polyphagous behavior and presumably high ecological tolerance it has successfully invaded other countries such as Korea and Japan. It was first detected in Pennsylvania in 2014, and within a few years it has rapidly spread to 13 counties in Pennsylvania. It has also spread to Virginia, New Jersey, Delaware (established populations), and by 2019, it was also detected in New York, Massachusetts, and Maryland. It is one of the most aggressive pests in Mid-Atlantic region: the range of woody tree species attacked by the lanternfly is extremely wide (over 70 woody host plants) and includes tree-of heaven, birch, maple, beech, oak, tuliptree, apple trees, grapes, and many other fruit, ornamental, and forest trees etc. [7,8].

Both adults and nymphs (four nymphal instars) cause severe plant damage by sucking phloem sap and excreting large volumes of a sugary substance, projectile honeydew [9]. Typically, nymphs ascend their host trees as soon as they hatch, they feed on leaves and branches, they frequently fall due to some environmental factors (e.g., wind), and then re-ascend the tree. As nymphs mature, their host plant range decreases, and they remain on host plants longer. They have a few preferred host plants at the adult stage (especially before laying eggs) [9]. Assessing and predicting host usage of the lanternfly has been challenging, and very little is known about the lanternfly association with its host plants at different developmental stages. Meanwhile, this information is very much needed for effective monitoring of the lanternfly on its host plants throughout the season.

Insect mouthparts and tarsal tips provide first and often primary contact with their host plants. Mouthparts of hemipterans (true bugs), *inter alia* leafhoppers and planthoppers, are highly modified for piercing plant tissue and sucking plant sap, and are extremely complex. The mouthparts that penetrate the plant are the stylets, and typical feeding behavior of true bugs after arriving on a host plant include (a) plant surface exploration, (b) penetration in plant tissue (stylet probing), (c) ingestion of plant fluid, and (d) termination of stylet probing [10]. The knowledge of the stylet morphology and morphology of the labium (the lower lip, modified to the tubular segmented appendage which houses the stylets) is instrumental in predicting the depth of stylet penetration and the intensity of plant damage. Previous studies on the morphology of the lanternfly mouthparts have focused on exploring chemoreceptors in the lanternfly mouthparts [11,12], as well as described the stylet and labium morphology in adults. However, to the best of our knowledge, the development of the morphological structures of the labium and stylets at each developmental stage of the lanternfly has yet to be studied.

It is also important for sap-feeders to be able to climb their host plants and firmly attach to plant surfaces. Many insects use specialized appendages for adhesion to plant and other surfaces. Insect tarsal claws and an arolium (an unpaired adhesive pad on the tarsal tips) play an important role in the attachment process. Previous studies showed that the structure of the arolium changes during the lanternfly growth, and its size is several times larger in adults than 1st-instar nymphs [9]. Though young nymphs can climb host plants, such as trees, their smaller arolia prevent them from firmly attaching to tree surfaces. They fall and feed on plants that they encounter while on the ground [9]. While nymphs are growing their arolia become stronger and falling-ascending cycle become longer [9]. The arolium was previously described for the spotted lanternfly adults [13]; the authors also observed that the adhesive properties of arolia in the lanternfly decreases with insect age due to wear. The morphology of the arolia in nymphs, as well as developmental changes in tarsal claws, however, have not been explored. Meanwhile, it is very important to evaluate changes in the morphology of the lanternfly tarsal tips during insect development as it will help better understand the lanternfly association with host plants, and specifically insect host plant preference and usage at each developmental stage.

To address these limitations, we focused on the following two objectives: (a) to assess changes in morphology of the lanternfly mouthparts (stylets and labium), and (b) to assess changes in morphology of the lanternfly tarsal tips (arolia and tarsal claws) at each developmental stage. The labium, stylets, and tarsal tips are the structures which are associated with primary contact of the lanternfly with its host plant, and which potentially facilitate the lanternfly successful host plant use. We assessed the developmental changes in these structures using both scanning electron microscopy and morphometric analysis. We expected these structures to undergo substantial morphological and morphometric changes throughout the lanternfly development which could potentially indicate the lanternfly association with certain host trees at each developmental stage.

## Materials and methods

To explore the morphology of the mouthparts and tarsal tips, a total of 70 individual insects (nine adults and 61 nymphs at various developmental stages: seven 1^st^-instars, seven 2^nd^-instars, 18 3^rd^-instars, and 29 4^th^-instars) were collected from 13 various host trees and dissected for microscopic observations. We then focused on morphological investigations of the labium, stylets, tarsal claws, and arolium using two approaches: (a) scanning electron microscopy, and (b) morphometric analysis (The protocol is available at http://dx.doi.org/10.17504/protocols.io.8tthwnn).

### Insect collecting and preserving

Nymphs and adults of the spotted lanternfly were collected from multiple locations in Berks County, PA in Summer-Fall, 2018. We collected second to fourth-instar nymphs in July, 2018; the insects were immediately preserved in 80% ethanol and transported to our laboratory at the University of Maryland. First-instar nymphs and adults preserved in 80%-ethanol were donated for this study by Dr. Greg Krawczyk’s lab (Pennsylvania State University, Fruit Research and Extension Center, Biglerville, PA). Both nymphs and adults were stored at 4°C until they were dissected.

### Dissection and tissue preparation

Individual insects at each developmental stage were placed on a microscope slide. Under the dissecting microscope (Zeiss, Germany) the head with the mouthparts was separated from the insect body, the labium was isolated and the stylets were exposed using a pair of fine tweezers from the micro dissecting kit (BioQuip Products Inc., Rancho Dominguez, CA, USA; micro dissecting kit, Cat. No. 4761). Similarly, the tarsus from one of the forelegs was separated using the micro slide tool kit (BioQuip Products Inc., Rancho Dominguez, CA, USA; micro slide tool kit, Cat. No. 4831).

### Morphometric measurements and statistical analysis

**Fig 1:**
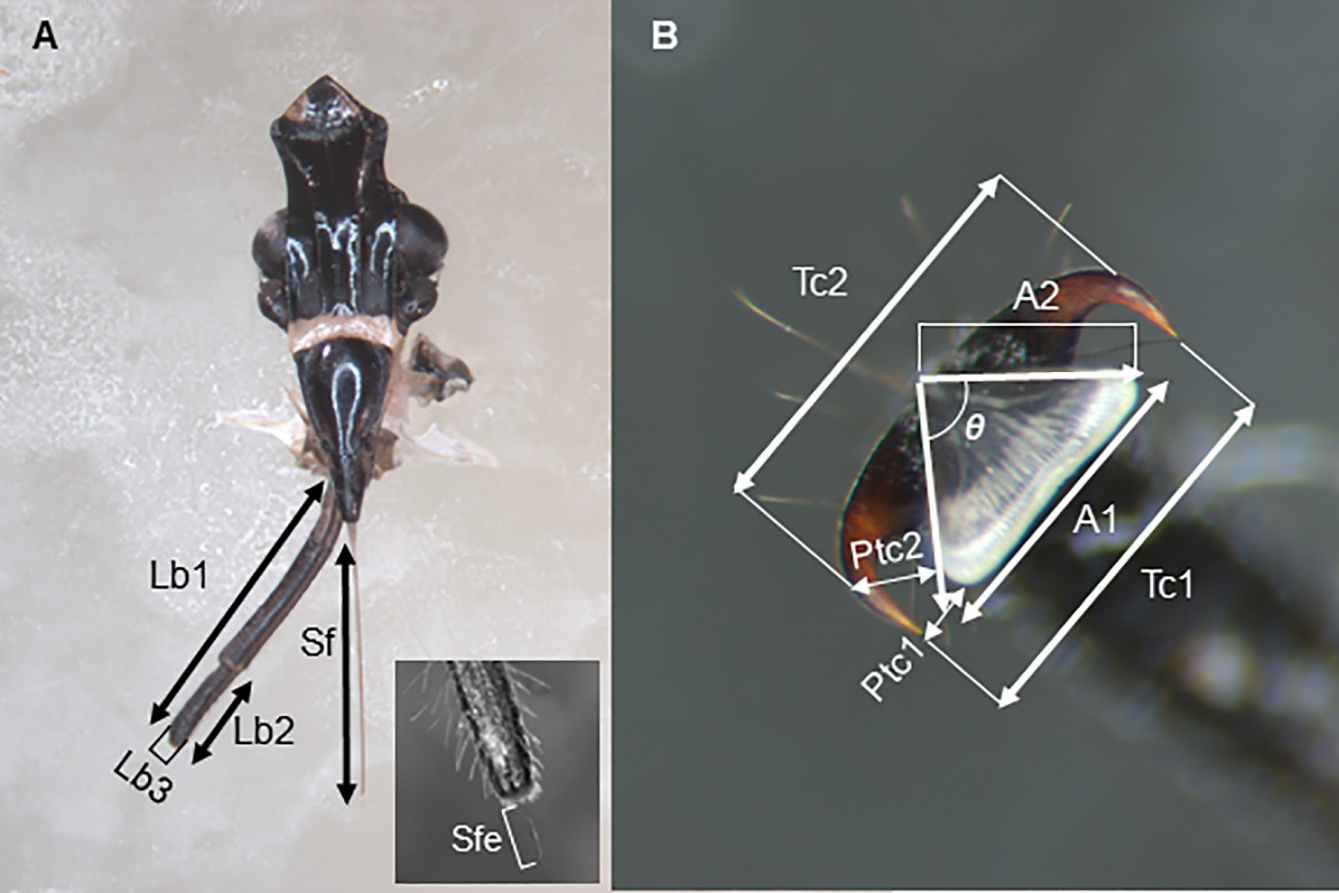
Morphometric characteristics measured for the labium, the stylet fascicle and the tarsal tip of *Lycorma delicatula*. (A) Labium and stylets. Lb1, distance from the labial tip to the base of the first labial segment; Lb2, distance from the labial tip to the base of the last labial segment; Lb3, maximum width of the last labial segment; Sf, distance from the tip of the stylet fascicle to the base of the stylets; Sfe, distance from the apex of stylet fascicle extended from labial tip to the labial tip. (B) Tarsal tip, the dorsal view. Tc1, distance between tarsal claw tips; Tc2, distance between bending centers of the external arcs of the tarsal claws; Ptc1, distance between the lateral margin of the arolium and tarsal claw tips; Ptc2, distance between the lateral margin of the arolium and bending centers of the external arcs of the tarsal claws; A1, the maximum anterior width of the arolium; A2, length of the lateral margin of the arolium; *ᶿ*, the angle between the lateral margins of the arolium.

The head with the mouthparts, as well as the labium, the stylet fascicle, and the dorsal view of the tarsal tip were photographed for each individual insect with a Zeiss Axio-Imager M1 using Zeiss ZEN imaging software (Carl Zeiss, Jena, Germany). Using these photographs, the following 12 morphometric characteristics were measured: (1) Distance from the labial tip to the base of the first labial segment; μm; (2) Distance from the labial tip to the base of the last labial segment; μm; (3) Maximum width of the last labial segment; μm; (4) Distance from the tip of the stylet fascicle to the base of the stylets; μm; (5) Distance from the apex of stylet fascicle extended from labial tip to the labial tip; μm; (6) Distance between tarsal claw tips from the dorsal view; μm; (7) Distance between bending centers of the external arcs of the tarsal claws from the dorsal view; μm; (8) Distance between the lateral margin of the arolium and tarsal claw tips from the dorsal view; μm; (9) Distance between the lateral margin of the arolium and bending centers of the external arcs of the tarsal claws from the dorsal view; μm; (10) The maximum anterior width of the arolium; μm; (11) Length of the lateral margin of the arolium; μm; and (12) The angle between the lateral margins of the arolium from the dorsal view; degrees (Fig 1, S1 Table, S2 Table). The isolated mouthparts and tarsi were then transferred back to 80%-ethanol for scanning electron microscopy.

Statistical analysis was conducted using multivariate analysis of variance (MANOVA) followed by one-way ANOVA (to analyze individual morphometric measurements of interest) with post hoc Tukey's HSD test to identify differences in morphometric characteristics between developmental stages; differences between males and females have not been investigated. Exponential and quadratic models were fitted to create the growth curves for each morphometric measurement. All analyses were conducted in R (R v.3.5.2)[14].

### Scanning electron microscopy (SEM) and image processing

Tissue fixation was done using the hexamethyldisilazane drying technique modified from Laforsh and Tollrian [15]. The mouthparts and tarsi were dehydrated by transferring them from 80% ethanol to 95%-ethanol for 10 min and then to 100%-ethanol, three times for 10 min. The specimens were then immersed in a graded series of 100%-ethanol and 100%-hexamethyldisilazane (HMDS), 2:1, 1:1, and 1:2 for 10 min each. Finally, the specimens were immersed in 100%-HMDS for three changes of 15, 30, and 45 min. After the last HMDS change, the specimens were just covered by fresh 100%-HMDS and they were moved to a vacuum desiccator for air drying at room temperature. The mouthparts and tarsi were then mounted to stubs and were coated with 10 nm of gold/palladium in a sputter coater. The specimens were then examined and imaged in Hitachi SU-3500 scanning electron microscope. Each photograph served as a reference for identification of morphological structures of the mouthparts and tarsal tips at each developmental stage of the lanternfly. We specifically focused on morphological analysis of (a) the labium and stylets, and (b) tarsal claws and arolia. Additionally, we explored the presence (or absence) of different types of labial sensilla at each developmental stage. We were particularly interested in finding and describing the bristle-like sensilla in late instars and adults. The bristle-like sensilla were found in fulgorid planthoppers only and not present in other species in Fulgormorpha. As a result, it has been suggested that they might be associated with bark feeding [11].

## Results

We investigated the external morphology of the mouthparts and tarsal tips using a total of 70 and 42 individual insects (respectively) at various developmental stages. Additionally, we explored differences (if any) in morphological structures of the mouthparts and tarsal tips between males and females. Using SEM images we primarily focused on the labium shape, number of segments, types of sensilla at the labial tip, presence or absence of any serrated ridges and protuberances at the apical end of the stylets, position and shape of the tarsal claws, and the arolium surface at each developmental stage of the lanternfly. Additionally, we used a total of 12 morphometric measurements to assess morphological changes in the labium, stylets, tarsal claws, and the arolium during the lanternfly development. We focused on detecting the differences in these morphometric characteristics among 1-4^th^ instar nymphs and adults only; we did not explore morphometric differences (if any) between males and females due to small sample sizes.

### Labium and labial tip

**Fig 2:**
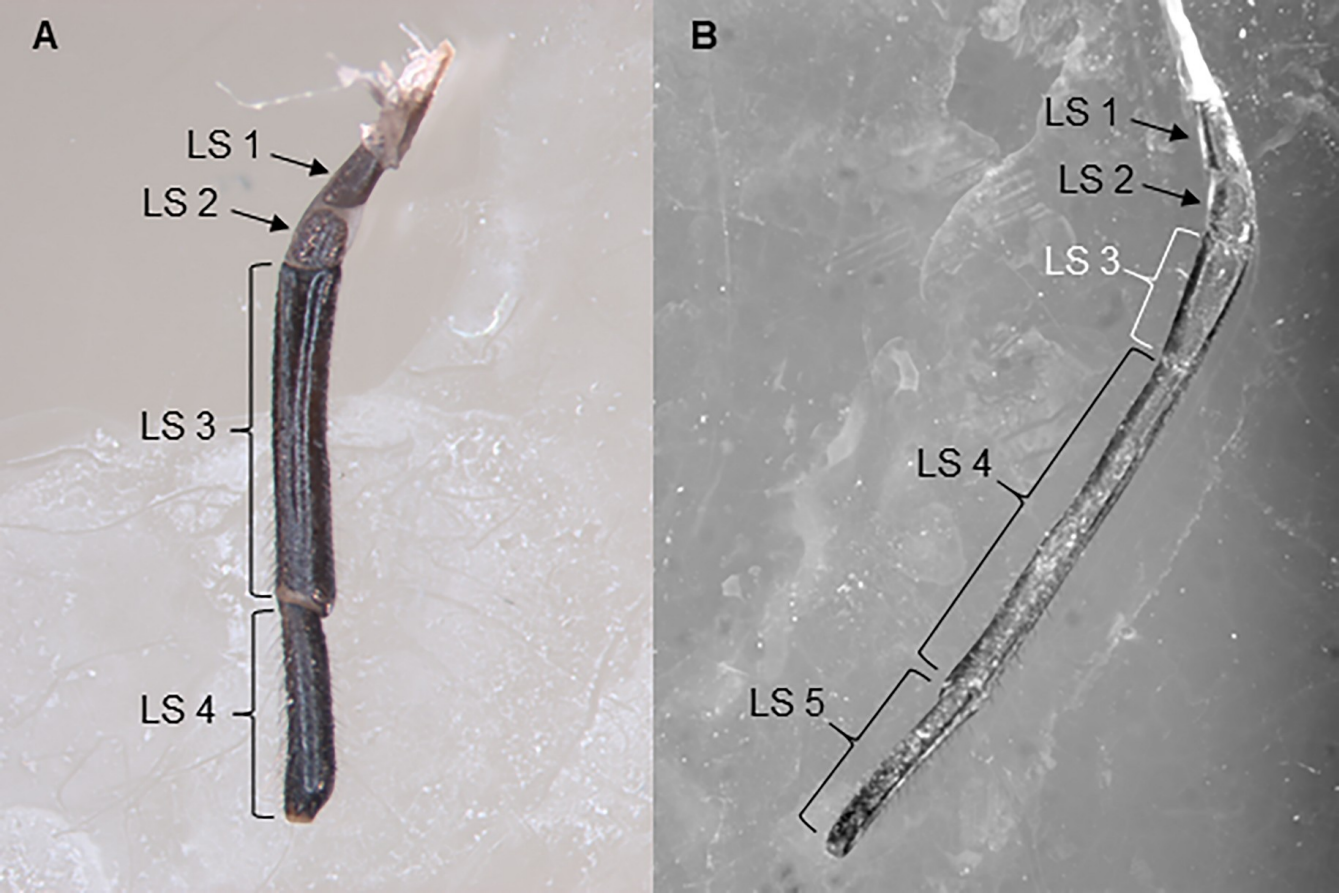
Labium and labial segments of *Lycorma delicatula* in nymphs and adults. (A) 4^th^ instar nymph; (B) Adult male. LS1-LS5, labial segments; LS1, LS2, LS3, and LS4 in nymphs correspond respectively to LS1, LS2, LS4, and LS5 in adults; LS3 in adults (white) is the 5^th^ (extra) segment.

The labium consists of four segments in 1-4^th^ instar nymphs and five segments in adults (Fig 2). The fifth segment in adults (segment LS3 in adults on Fig 2B) has somewhat conical shape and it is located near the middle point of the labium, between two basal labium segments (LS1 and L2 in both nymphs and adults; Fig 2A and 2B) and two last labium segments LS4 and LS5 (LS3 and LS4 in nymphs on Fig 2A). The length and maximum width of this extra segment in adults are 1574 ±363 μm and 733±96 μm respectively.

**Fig 3:**
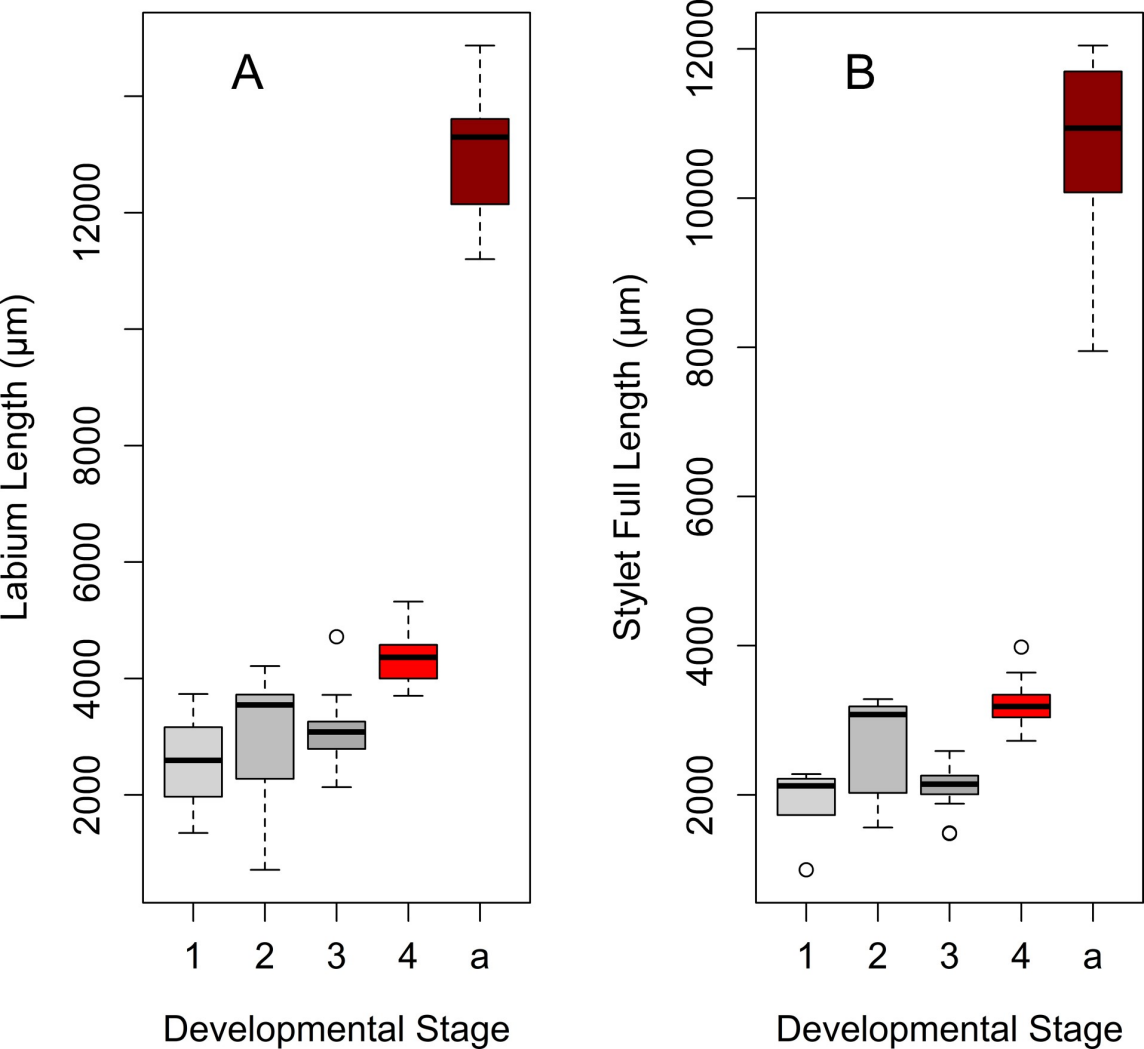
Labium and stylet length of *Lycorma delicatula* across developmental stages. (A) Labium length changes. (B) Stylet length changes. Axis labels: 1–4, 1^st^-4^th^ instar nymphs; a, adults.

**Fig 4:**
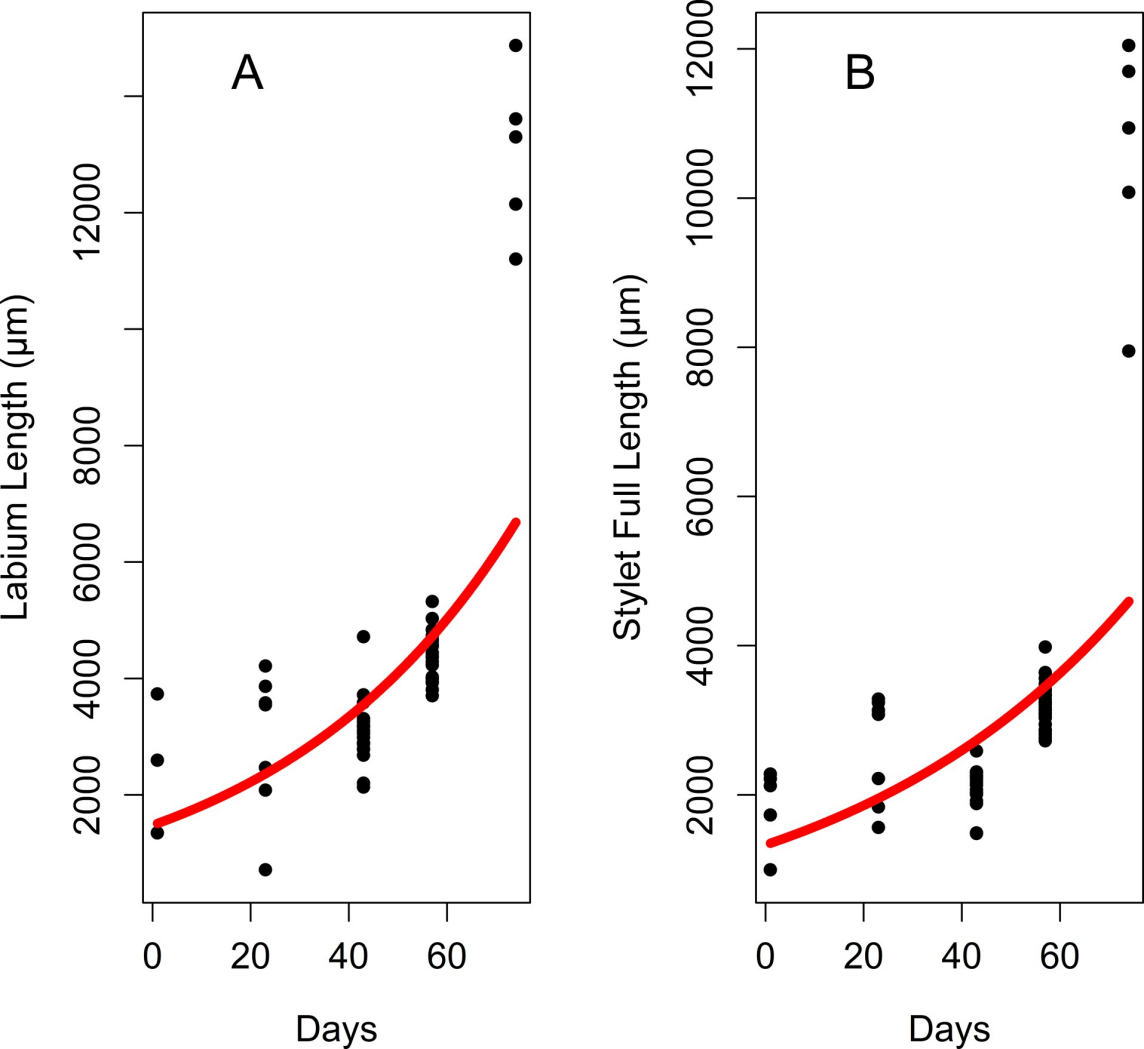
Growth curves for the labium and stylet length during the lanternfly development. (A) Labium length changes, exponential model (*y* = 1465.57*e*^0.02*x*^, R^2^ = 0.49). (B) Stylet length changes, exponential model (*y* = 1326.1*e*^0.01*x*^, R^2^ = 0.43). Axis labels: Day, days of the lanternfly development; day 0, hatching of the 1^st^ nymphal instar; day 74, appearance of the adults (based on dates reported in Dara et al. [8]).

**Fig 5:**
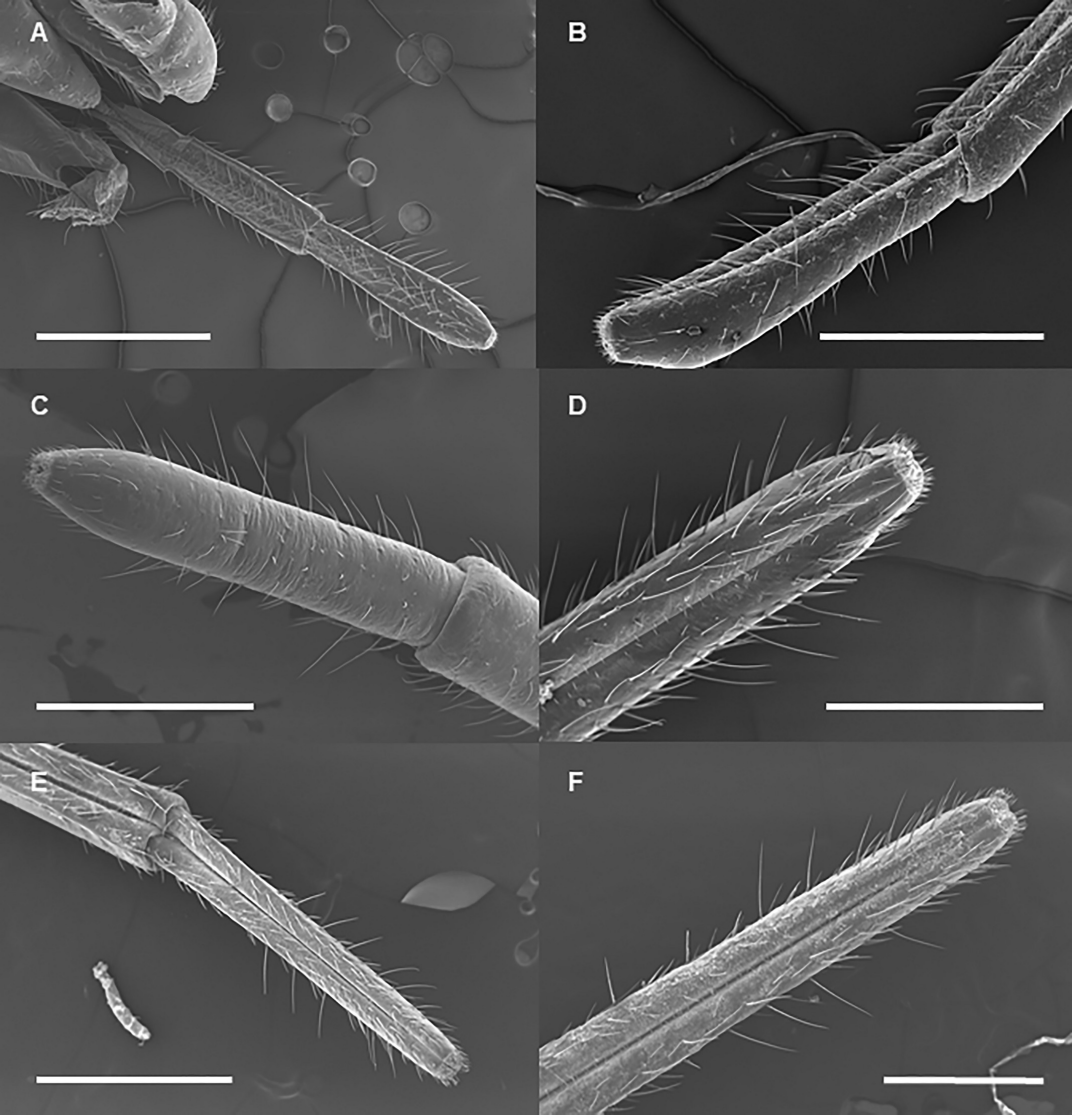
SEM of the last segment of labium of *Lycorma delicatula* at each developmental stage. (A) First instar nymph. (B) Second instar nymph. (C) Third instar nymph. (D) Fourth instar nymph. (E) Adult female. (F) Adult male. Bars: (A), (C), (D), and (F) = 500 μm; (B) = 400 μm; (E) = 1 mm.

**Fig 6:**
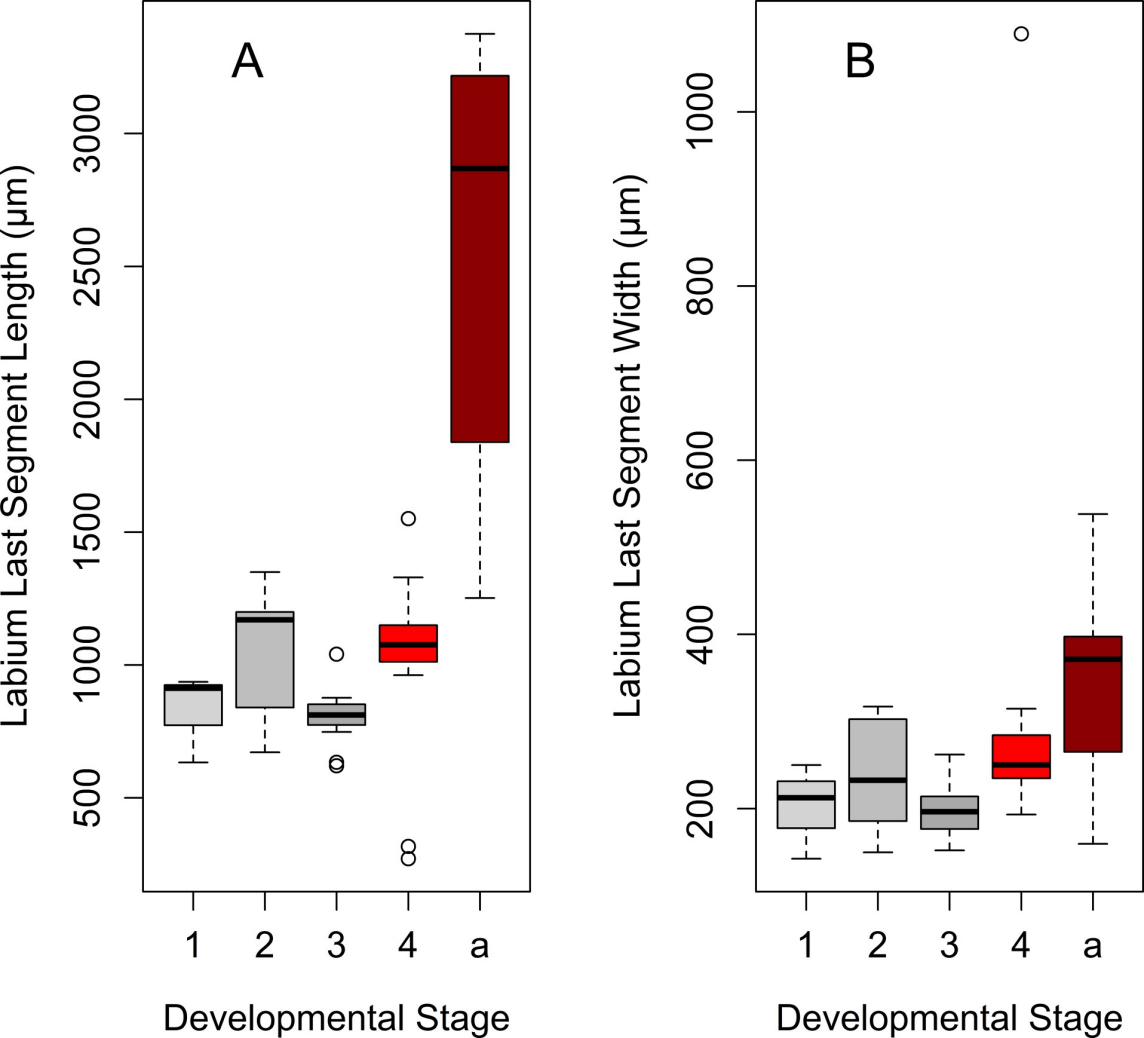
Size changes in the last labial segment of *Lycorma delicatula* across developmental stages. (A) Last segment length. (B) Last segment width. Axis labels: 1–4, 1^st^-4^th^ instar nymphs; a, adults.

**Fig 7:**
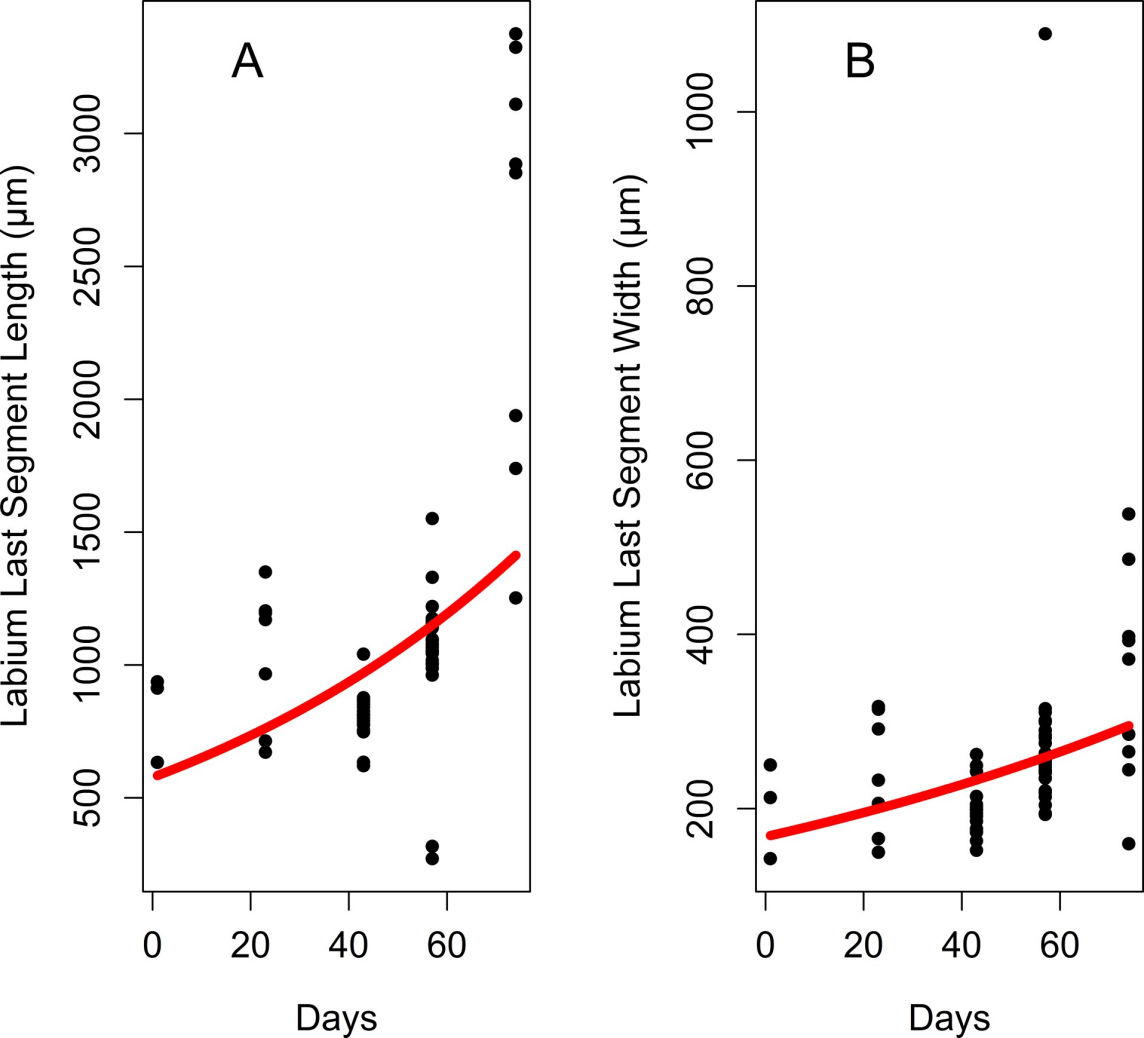
Growth curves for the last labial segment during the lanternfly development. (A) Last segment length, exponential model (*y* = 572.49*e*^0.01*x*^, R^2^ = 0.21). (B) Last segment width, exponential model (*y* = 167.33*e*^0.007*x*^, R^2^ = 0.16). Axis labels: Day, days of the lanternfly development; day 0, hatching of the 1^st^ nymphal instar; day 74, appearance of the adults (based on dates reported in Dara et al. [8]).

Labium length differs significantly among developmental stages, exponentially increasing by 4^th^-instar nymph and the adult stage (Table 1, Fig 3, Fig 4). The last labial segment (at the end the labium) is cylindrical at each developmental stage (Fig 5). Its length also differs significantly among the developmental stages and increases exponentially from the 1^st^ nymphal instar to the adult stage (Fig 6A, Fig 7A); while the difference in its width is significant only between adults and 3^th^–instar nymph (Table 1, Fig 6B, Fig 7B).

**Table 1:** The morphometric data of the labium and stylet fascicle of *Lycorma delicatula* at each developmental stage (mean±SE). LL, labium length; LSL, last labial segment length; LSW, last labial segment width; SL, stylets–full length; SEL, stylets–length of the exposed part. Different letters indicate significant differences (*P<0*.*05*).

Stage	LL (μm)	n	LSL (μm)	n	LSW (μm)	n	SL (μm)	n	SEL (μm)	n
**1**^**st**^ **instar**	2558±689^a^	3	827±97 ^a^	3	202±32 ^a^	3	1867±238 ^a^	3	N/A	N/A
**2**^**nd**^ **instar**	2923±467 ^a^	7	1039±99 ^a^	7	239±26 ^a^	7	2621±275 ^ab^	7	N/A	N/A
**3**^**rd**^ **instar**	3090±134 ^a^	18	806±22 ^a^	18	199±7 ^ab^	18	2090±65 ^a^	18	105±10^a^	7
**4**^**th**^ **instar**	4343±73 ^b^	29	1051±46 ^a^	29	283±31 ^a^	29	3194±53 ^b^	29	216±27^b^	19
**Adults**	13025±629 ^c^	5	2559±284 ^b^	8	349±40 ^ac^	9	10542±731 ^c^	5	523±72	2
**Adult female**	N/A	N/A	2100±542	3	321±113	3	N/A	N/A	N/A	N/A
**Adult male**	12957±808	4	2835±295	5	363±35	6	10166±810	4	523±72	2

**Fig 8:**
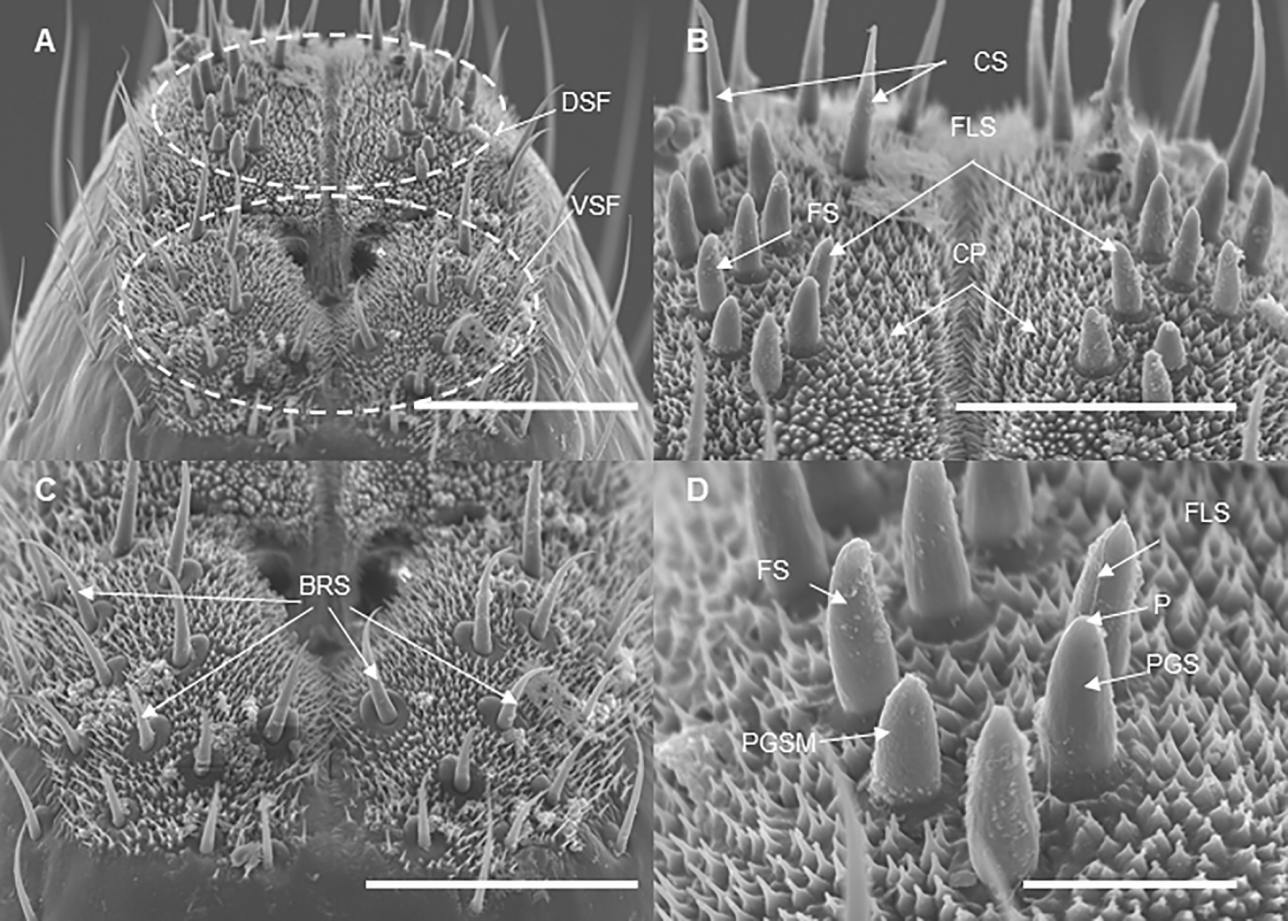
SEM of the labial tip of *Lycorma delicatula* (Third instar nymph). (A) Sensory fields. DSF, dorsal sensory field; VSF, ventral sensory field. (B) and (D) Dorsal sensory field. CS, clavate sensilla; FS, forticate sensilla, FLS, finger-like sensilla; CP, cuticular process; PGSM, multiporous peg sensilla; PGS, peg sensilla; P, pore. (labeled following Hao et al. [12]). Bars: (A) = 50 μm; (B) = 30 μm; (C) = 40 μm; (D) = 10 μm.

**Fig 9:**
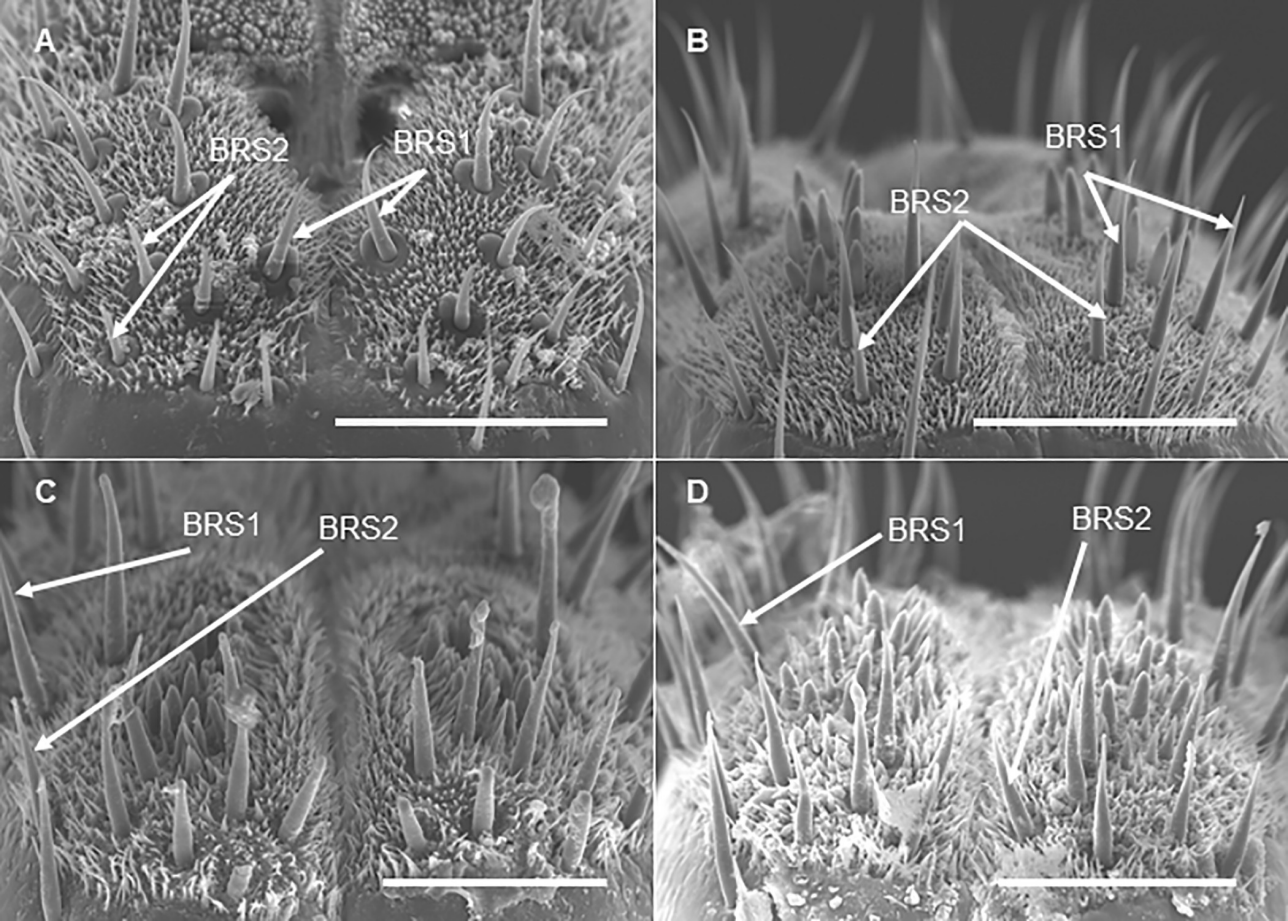
SEM of bristle-like sensilla on the labial tip of *Lycorma delicatula* nymphs and adults. (A) Third instar nymph, dorsal sensory field. (B) Fourth instar nymph, ventral sensory field. (C) Adult female, ventral sensory field. (D) Adult male, ventral sensory field. BRS1, long bristle-like sensilla; BRS2, short bristle-like sensilla. Bars: (A), (B), (C), and (D) = 50 μm.

The surface of the labium carries numerous sensilla at each developmental stage. At each developmental stage, the tip of the labium is divided into two lobes by the labial groove: each lobe carries one ventral and one dorsal sensory field which have numerous sensilla (Fig 8A). Sensilla are located asymmetrically and surrounded by cuticular processes (Fig 8B). We observed six different morphological types of sensilla: bristle-like sensilla (BRS; two types: short and long), clavate sensilla (CS), forticate sensilla (FS), peg sensilla (PGS), multiporous sensilla (PGSM), and finger-like sensilla (FLS) (Fig 8B–8D). At each developmental stage, all the types of the sensilla are present on the dorsal fields while BRS are observed on both dorsal and ventral sensory fields (Table 2). The total number of BRS on the ventral fields (based on data recorded for adults and 3^rd^ and 4^th^ nymphal instars), however, are significantly higher than that on the dorsal fields (ANOVA: *F*_(1,10)_ = 20.77, *P* = 0.001) (Table 3, Fig 9). The length of BRS at these developmental stages ranges from 19.3–22.8 μm (short BRS) and from 25.6–28.9 μm (long BRS); whereas their basal width ranges 2–4 μm, in both long and short BRS (Table 3).

**Table 2:** Labial sensilla types, their presence ("+") or absence ("−") on each sensory field at each developmental stage of *Lycorma delicatula*.

Developmental stage	Bristle-like sensilla (+/−)		Clavate sensilla, Forticate sensilla, Peg sensilla, Multiporous peg sensilla, Finger-like sensilla; (+/−)	
	Dorsal	Ventral	Dorsal	Ventral
**1**^**st**^ **instar**	+	+	+	−
**2**^**nd**^ **instar**	+	+	+	−
**3**^**rd**^ **instar**	+	+	+	−
**4**^**th**^ **instar**	+	+	+	−
**Adult female**	+	+	+	−
**Adult male**	+	+	+	−

**Table 3:** The total number and size of bristle-like sensilla (BRS) in 3^rd^-4^th^ instar nymphs and adults of *Lycorma delicatula* (mean±SE). DSF, dorsal sensory field; VSF, ventral sensory field.

Developmental stage	BRS total number		BRS length, (μm)		BRS basal width, (μm)	
	DSF	VSF	Long BRS	Short BRS	Long BRS	Short BRS
**3**^**rd**^ **instar**	12±1	25±1	25.6±1.5	19.3±0.6	2±0.2	2.2±0
**4**^**th**^ **instar**	18.5±0.5	21.5±1.5	27.6±28.9	21.2±1.3	3.3±0.1	2.5±0.1
**Adults**	16.5±1.5	22±1	28.9±0.9	22.8±0.9	4.8±0.2	4.2±0.2

### Stylets

**Fig 10:**
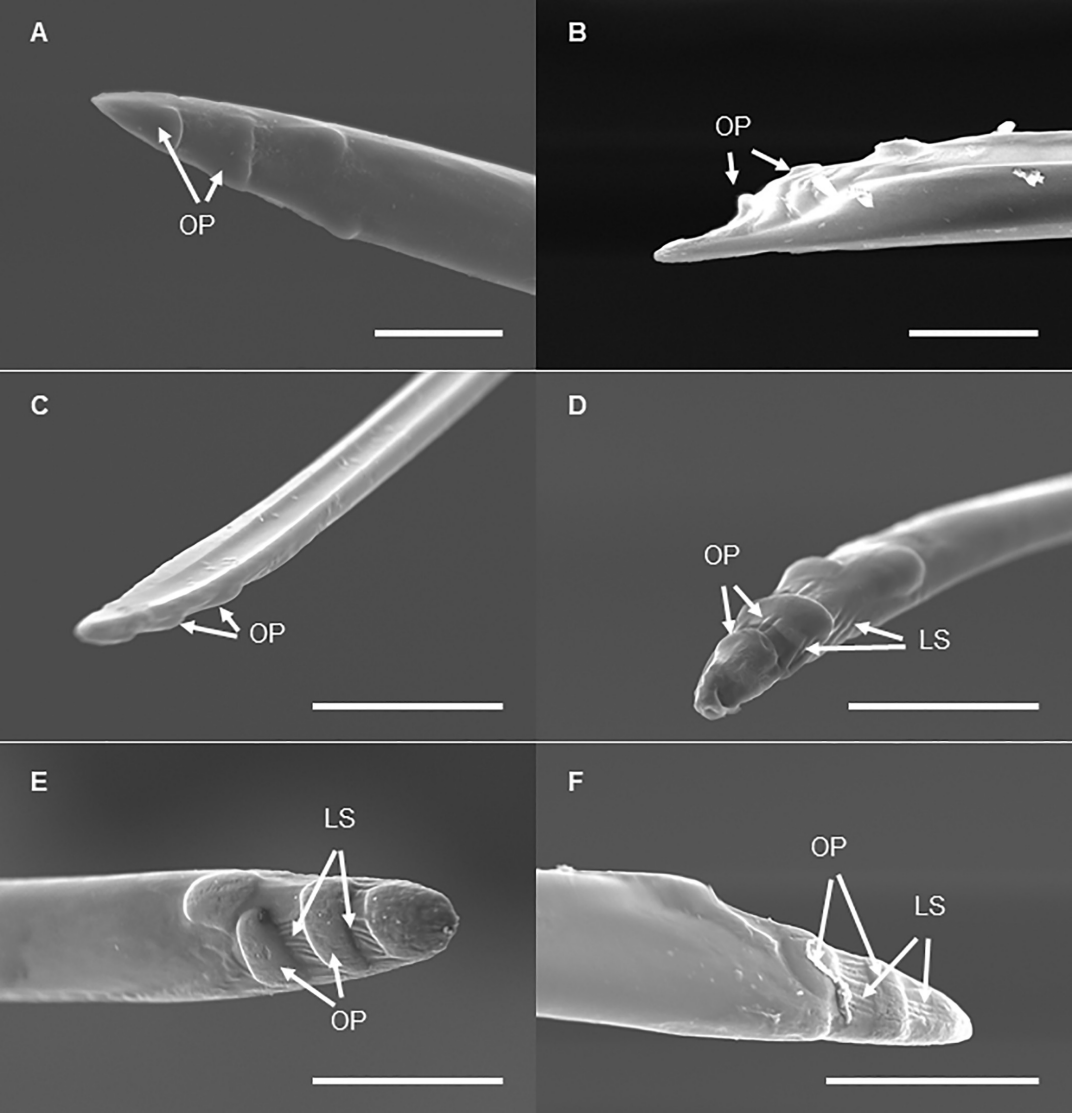
SEM of the mandibular stylets of *Lycorma delicatula* at each developmental stage. (A) First instar nymph. (B) Second instar nymph. (C) Third instar nymph. (D) Fourth instar nymph. (E) Adult female. (F) Adult male. OP, oval prominences; LS, longitudinal striations. Bars: (A) and (B) = 10 μm; (C) and (D) = 30 μm, (E) and (F) = 50 μm.

**Fig 11:**
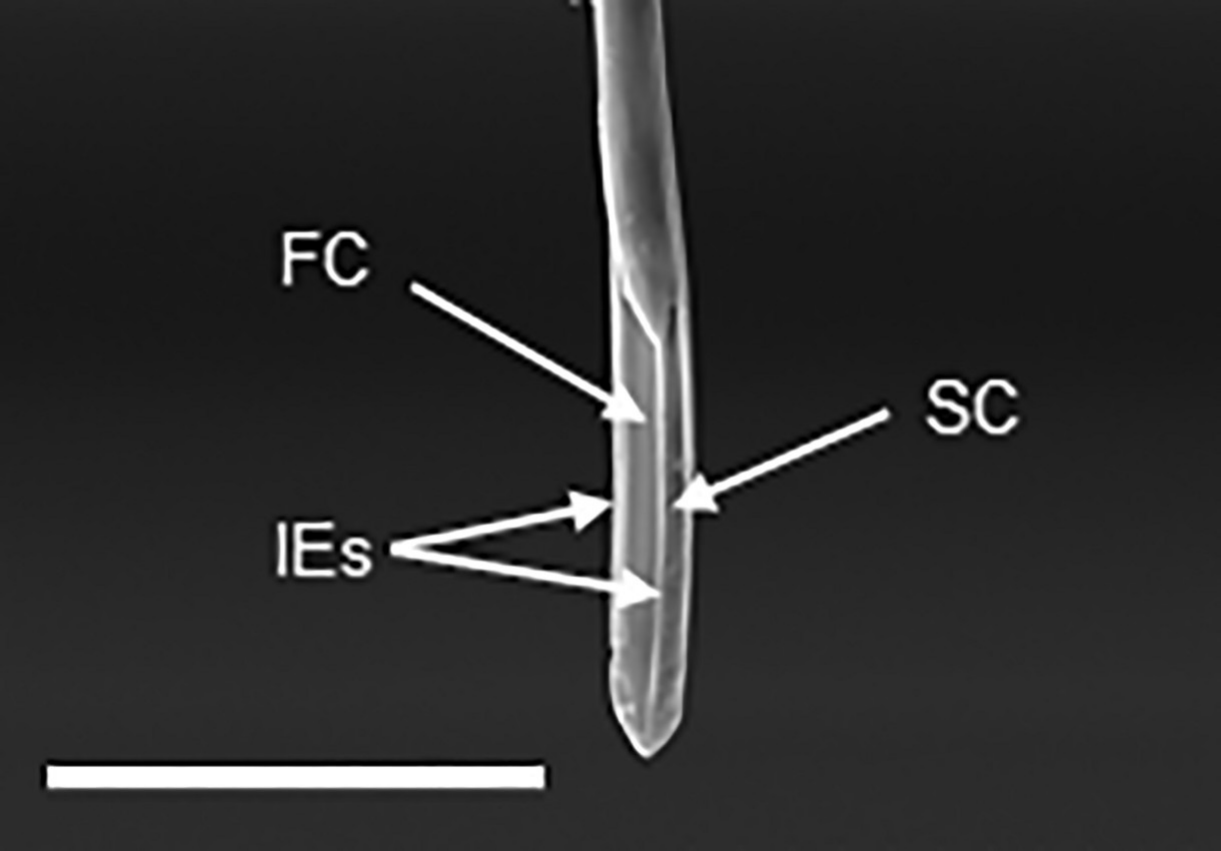
SEM of the maxillary stylets of *Lycorma delicatula* (second instar nymph). FC, food canal; SC, salivary canal; ICs, interlocking canals (labeled following Hao et al. [12]). Bar: 40 μm.

On the dorsal surface of the labium, along all of its length, the labial grove contained mandibular and maxillary stylets. At each developmental stage, each mandibular stylet possesses four indentations (oval prominences) on the outer surface at the apical region (Fig 10). The maximum diameter of the prominences varies from 3.8±0.9 μm (in 1^st^ nymphal instars) to 11.4±1.1 μm (in 4^th^ nymphal instars) and 21.6±2.1 μm in adult females and 14.9±2.1 μm in adult males. Longitudinal striations between the oval prominences are also observed at the apical region of the mandibular stylets in 4th instar nymphs and adults (Fig 10D–10F); and they are not present in 1^st^-3^rd^ instar nymphs (Fig 10A–10C). Most of such longitudinal striations are located between the oval prominences at the apical region. The rest of the outer surface and the entire inner surface of the mandibular stylets is smooth. Maxillary stylets are morphologically similar across all the developmental stages. These stylets have smooth outer surface throughout their length. In the inner surface, we observed the food canal, salivary canal (labeled following Hao et al. [12]) and two interlocking edges (Fig 11).

The length of the stylet fascicle significantly differs among the developmental stages and exponentially increases by 4^th^ instar nymphs and adults (Table 1, Fig 3B and Fig 4B). We have also found that the length of the part of the stylet fascicle which protruded from the labial tip was larger in 4th instar nymphs compared to that in 3^rd^ instars (data for this comparison were available for 3 and 4^th^ instars only) (Table 1).

### Tarsal tip: Tarsal claws and arolium

**Fig 12:**
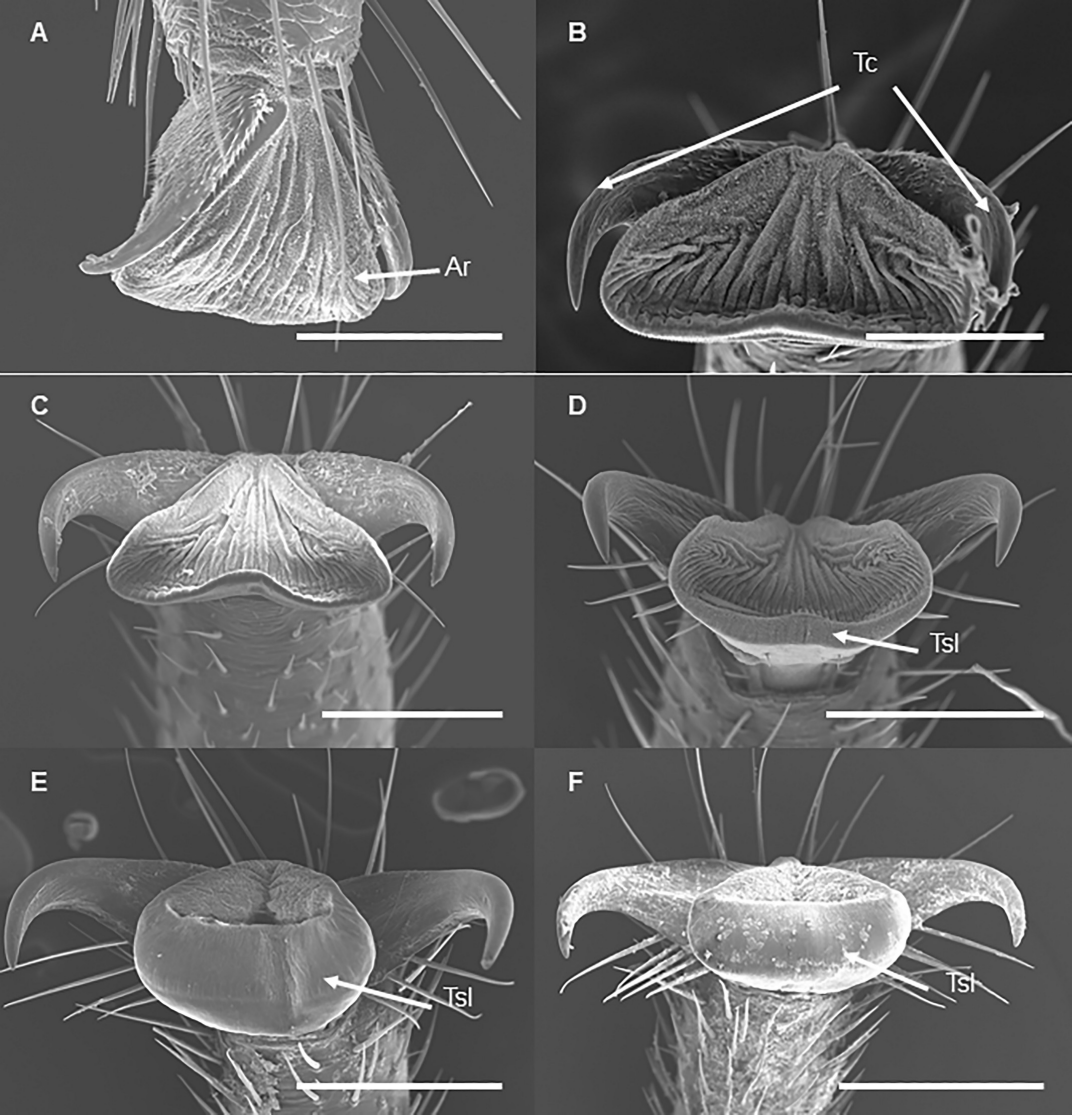
SEM of the tarsal tips of the forelegs of *Lycorma delicatula* at each developmental stage. (A) First instar nymph. (B) Second instar nymph. (C) Third instar nymph. (D) Fourth instar nymph. (E) Adult female. (F) Adult male. Ar, arolium; Tc, tarsal claw; Tsl, terminal sticky lip. Bars: (A) and (B) = 100 μm; (C) = 200 μm; (D) = 300 μm, (E) and (F) = 400 μm.

**Fig 13:**
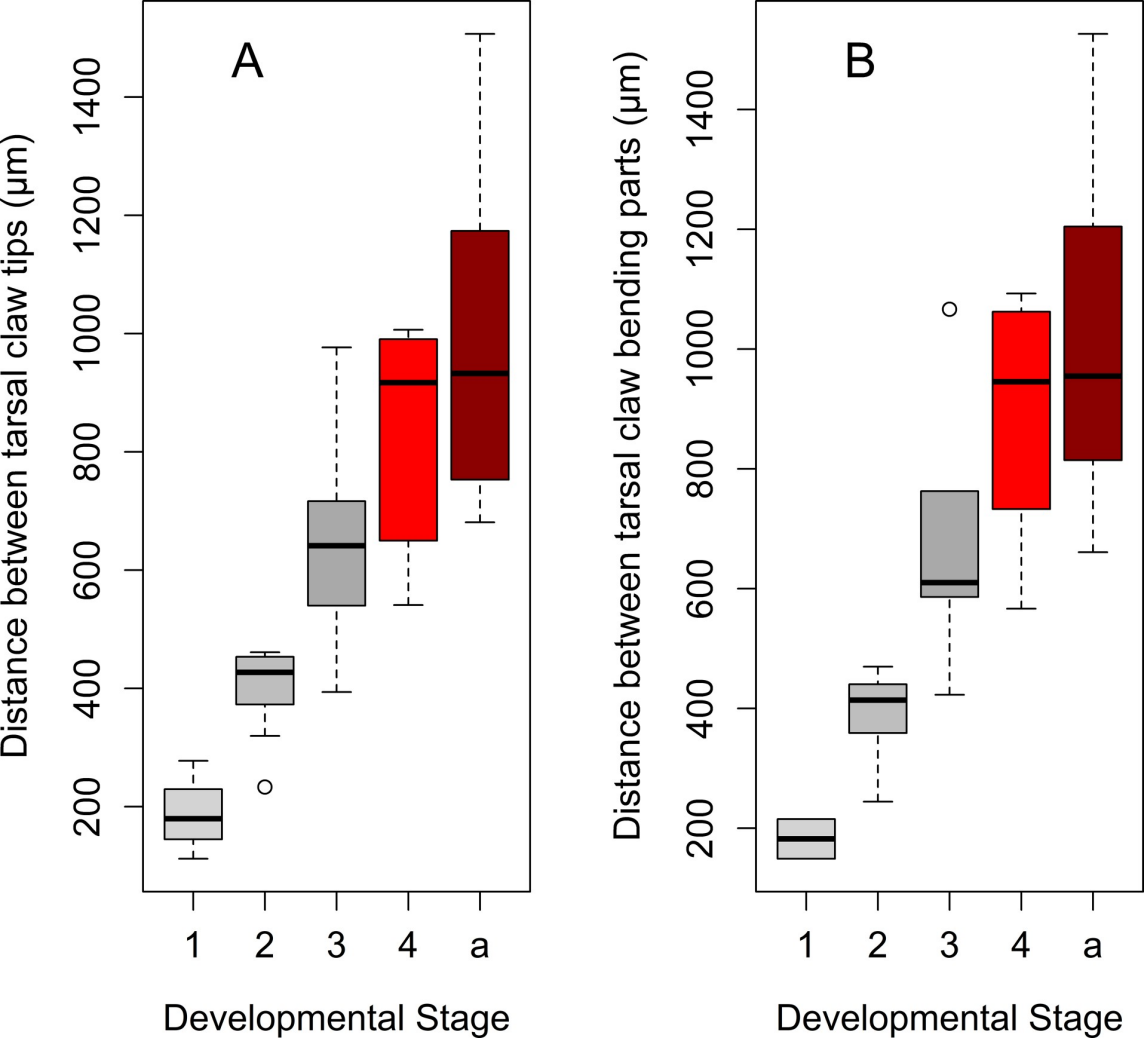
Distance between the tarsal claws of the forelegs of *Lycorma delicatula* across developmental stages. (A) Distance between tarsal claw tips. (B) Distance between bending centers of the external arcs of the tarsal claws. Axis labels: 1–4, 1^st^-4^th^ instar nymphs; a, adults.

**Fig 14:**
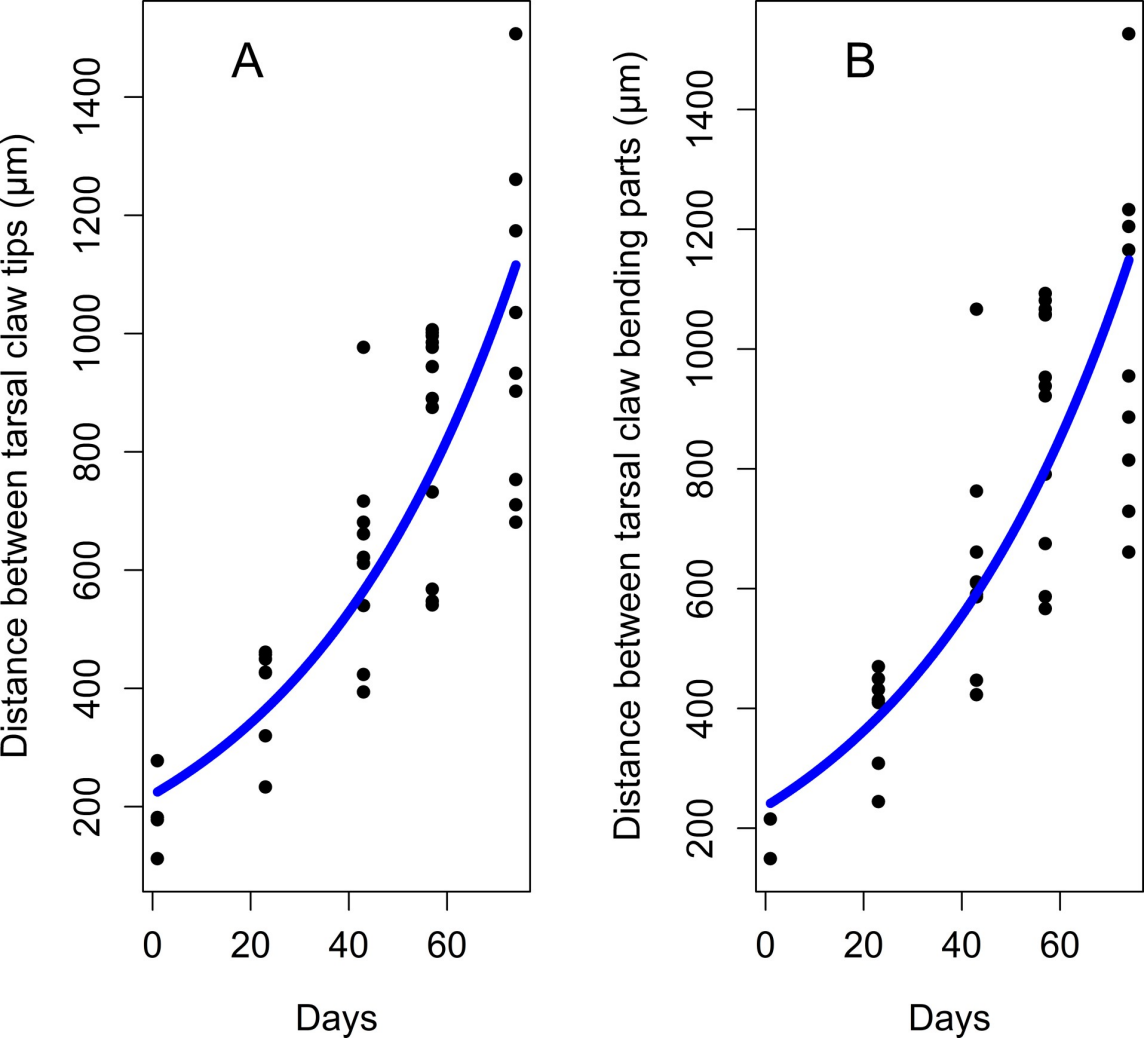
Growth curves for the distance between the tarsal claws of the forelegs during the lanternfly development. (A) Distance between tarsal claw tips, exponential model (*y* = 219.2*e*^0.02*x*^, R^2^ = 0.72). (B) Distance between bending centers of the external arcs of the tarsal claws, exponential model (*y* = 235.09*e*^0.02*x*^, R^2^ = 0.69). Axis labels: Day, days of the lanternfly development; day 0, hatching of the 1^st^ nymphal instar; day 74, appearance of the adults (based on dates reported in Dara et al. [8]).

The tarsal tip of a foreleg in each developmental stage carries two equal tarsal claws and an adhesive pad, arolium, located between tarsal claws (Fig 1B). We observed that in late instars (3^rd^ and 4^th^) and adults the tarsal claws are more spread out, while in the early nymphal instars (1^st^ and 2^nd^) the claws are located very close to the arolium (Fig 12). These observations were supported by comparisons of morphometric characteristics of tarsal claws and arolium: the distance between tarsal claw tips, as well as the distance between bending centers of the tarsal claws, differ significantly among the stages and exponentially increase by the adult stage (Table 4, Fig 13, and Fig 14). Interestingly, the distances between tarsal claws (both tips and bending centers) and arolium margin were significantly larger in adults only (Table 4).

**Table 4:** The morphometric data of the tarsal claws of *Lycorma delicatula* at each developmental stage (mean±SE). TCT, distance between tarsal claw tips; TCB, distance between tarsal claw bending parts; TCA, distance between tarsal claw tips and arolia, TBA, distance between tarsal claw bending parts and arolia. Different letters indicate significant differences (*P<0*.*05*).

Stage	TCT (μm)	n	TCB (μm)	n	TCA (μm)	n	TBA (μm)	n
**1**^**st**^ **instar**	187±34^a^	4	182±33	2	20±3	2	-3±4	2
**2**^**nd**^ **instar**	396±33^ad^	7	390±31^ab^	7	35±5 ^a^	7	32±4 ^a^	7
**3**^**rd**^ **instar**	660±62^cd^	10	682±71^bc^	10	80±8 ^a^	10	91±12 ^a^	10
**4**^**th**^ **instar**	839±55^b^	12	899±57^c^	12	95±32 ^a^	12	125±30 ^a^	12
**Adults**	995±93^b^	9	1019±94^dc^	9	201±26 ^b^	9	213±25 ^b^	9
**Adult female**	789±75	3	810±85	3	156±39	3	166±43	3
**Adult male**	1098±114	6	1124±115	6	224±31	6	237±27	6

**Fig 15:**
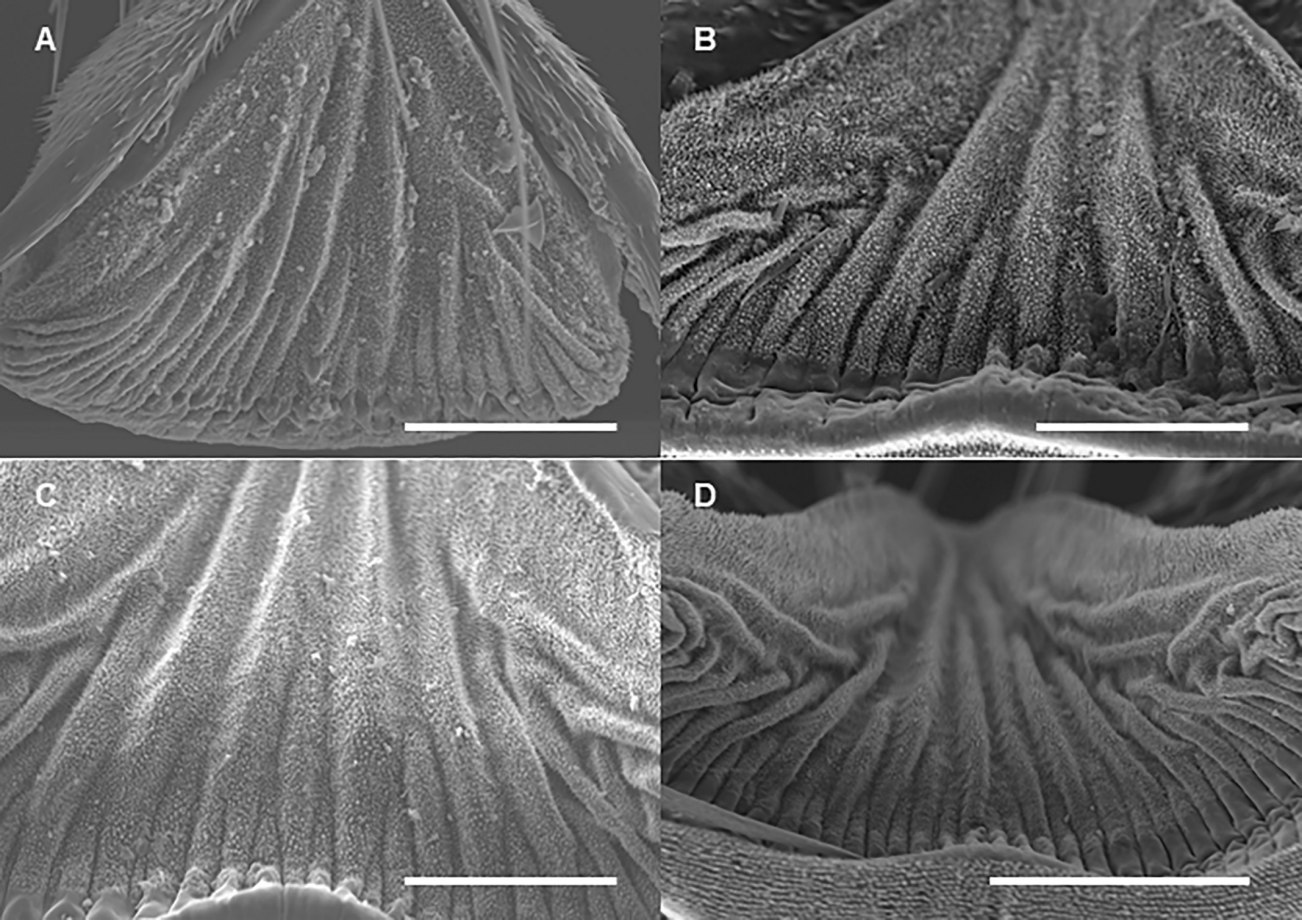
SEM of the arolium surface of the forelegs of *Lycorma delicatula* at each nymphal stage. (A) First instar nymph. (B) Second instar nymph. (C) Third instar nymph. (D) Fourth instar nymph. Bars: (A), (B), and (C) = 50 μm; (D) = 100 μm.

**Fig 16:**
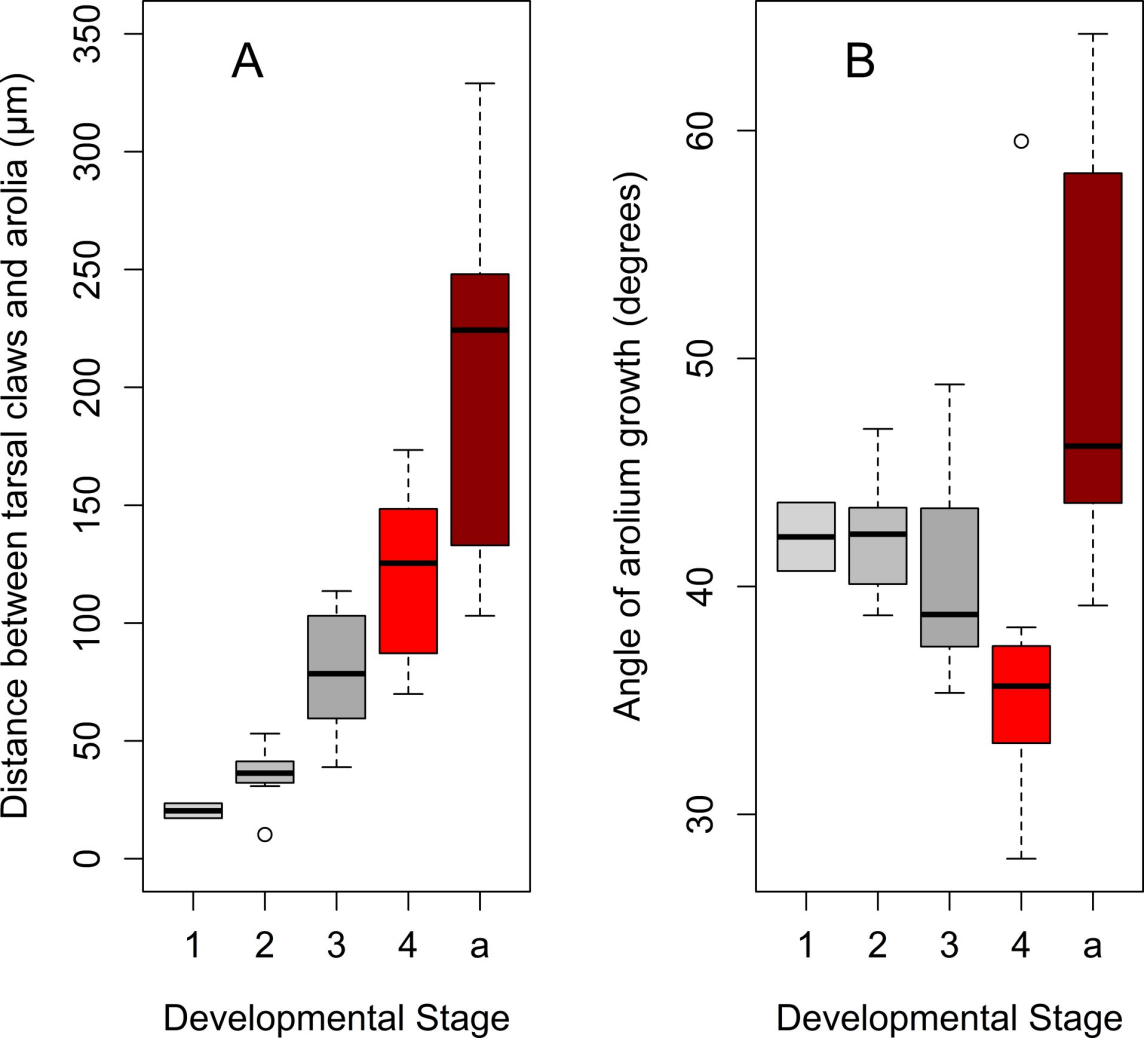
Distance between tarsal claws and arolium, and the angle of the arolium growth. (A) Distance between tarsal claws and arolium. (B) Angle of the arolium growth. Axis labels: 1–4, 1^st^-4^th^ instar nymphs; a, adults.

**Fig 17:**
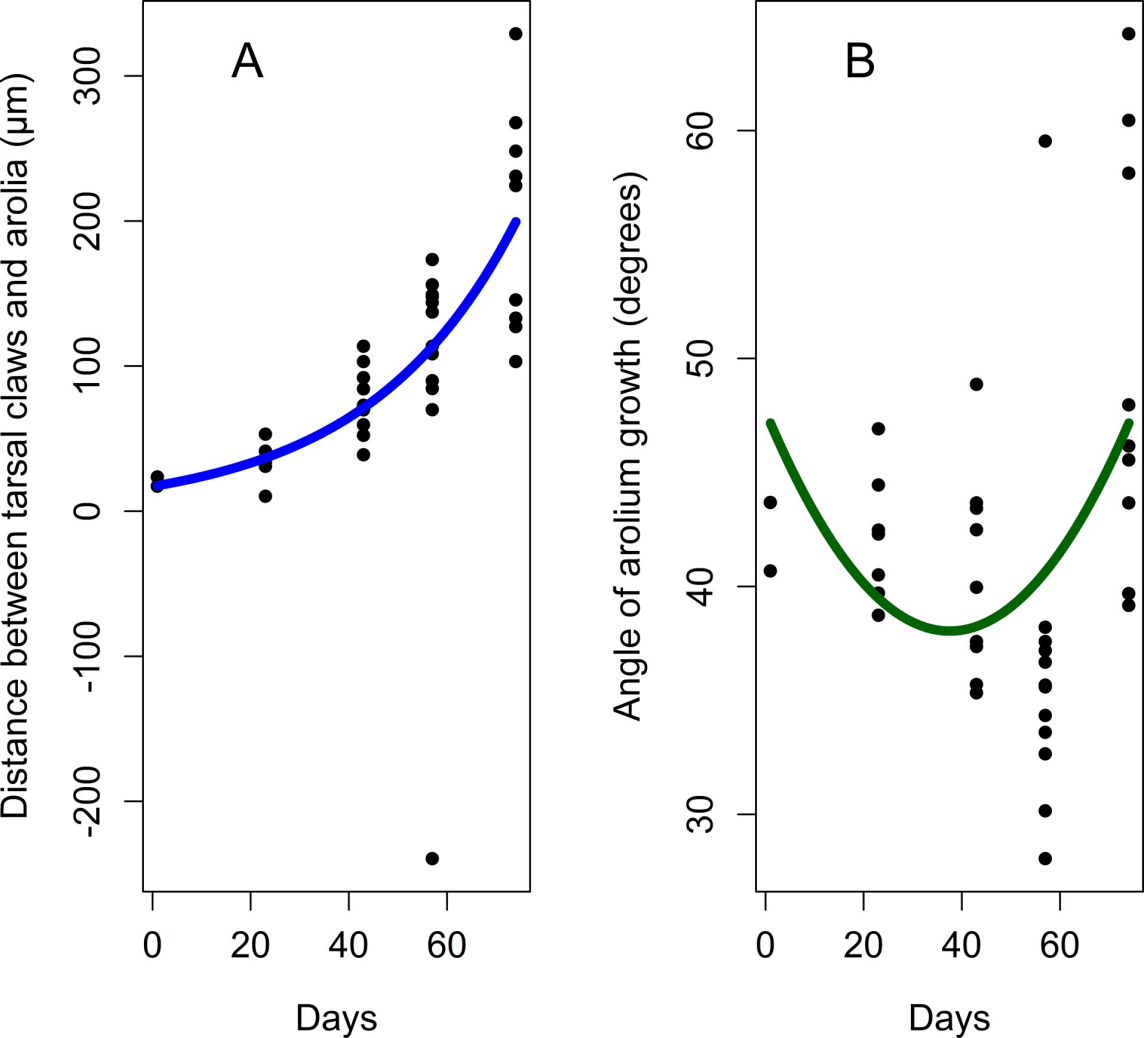
Growth curves for distance between tarsal claws and arolium, and the angle of the arolium growth during the lanternfly development. (A) Distance between tarsal claws and arolium, exponential model (*y* = 17.11*e*^0.03*x*^, R^2^ = 0.76). (B) Angle of the arolium growth, quadratic model (*y* = 47.67–0.51*x* + 0.007 *x*^2^, R^2^ = 0.16). Axis labels: Day, days of the lanternfly development; day 0, hatching of the 1^st^ nymphal instar; day 74, appearance of the adults (based on dates reported in Dara et al. [8]).

Arolia are fully developed at each developmental stage. Arolium base width, as well as the length of the lateral margin, increased by 4^th^ instar and they do not change in adults (Table 5). Following Frantsevich et al. [13], we observed that the dorsal surface of the arolium forms wrinkles which are more evident in adults, especially when the arolium is not completely spread out (Fig 12, Fig 15, and S2 Fig). The angle of arolium growth is mostly acute at all the nymphal stages; it is not different in 1-3th instars suggesting symmetrical growth in all directions during these stages. However, it becomes significantly narrower in 4^th^ instars, and then significantly increases and become obtuse in adults (Table 5, Fig 16B, Fig 17B, and S3 Fig).

**Table 5:** The morphometric data of the arolia of *Lycorma delicatula* at each developmental stage (mean±SE). AAG, angle of arolium growth; AAW, the anterior width of the arolium, ASL, arolia side length. Different letters indicate significant differences (*P<0*.*05*).

Stage	AAG (degrees)	n	AAW (μm)	n	ASL (μm)	n
**1**^**st**^ **instar**	42±2^a^	4	189±42	2	140±27	2
**2**^**nd**^ **instar**	42±1 ^a^	7	326±25^a^	7	245±21^a^	7
**3**^**rd**^ **instar**	40±1 ^a^	10	500±49^ac^	10	393±42^ac^	10
**4**^**th**^ **instar**	37±2 ^b^	12	650±50^bc^	12	571±60^bc^	12
**Adults**	49±3 ^c^	9	593±52^bc^	9	403±41^ab^	9
**Adult female**	48±6	3	478±5	3	333±34	3
**Adult male**	50±4	6	651±66	6	437±56	6

### Tarsal tip and labium allometry

**Fig 18:**
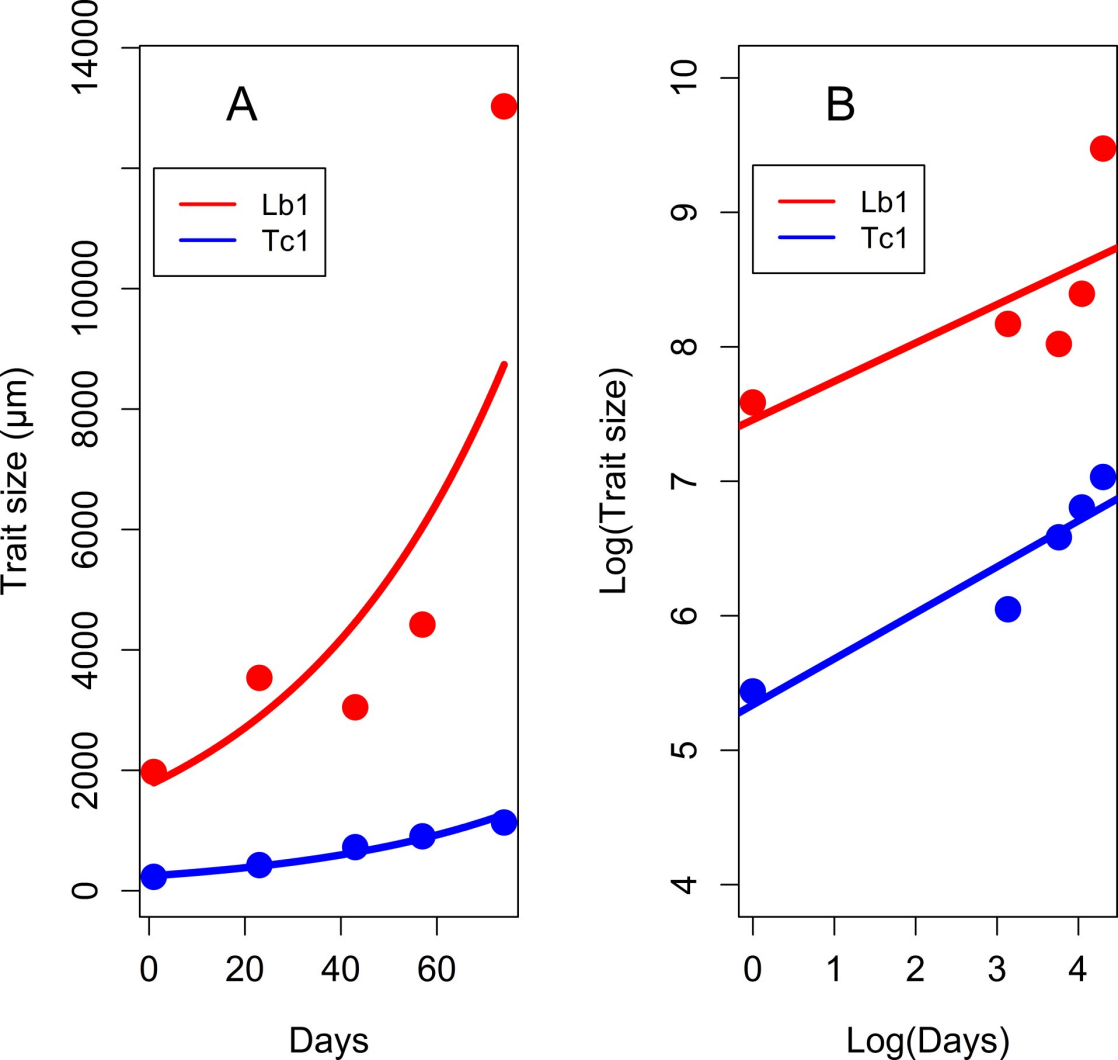
The allometric relationship between the labium length and the distance between the tarsal claws relative to the lanternfly developmental stages. (A) Original growth curves for the labium length (Lb1) and the distance between the tarsal claw tips (Tc1) (Lb1: *y* = 1465.57*e*^0.02*x*^; Tc1: *y* = 219.2*e*^0.02*x*^). (B) Growth curves for the labium length (Lb1) and the distance between the tarsal claw tips (Tc1) plotted on a log-log scale (Lb1: ln(*y*) = 7.46+0.29ln(*x*); Tc1: ln(*y*) = 5.34+0.34ln(*x*)). Axis labels: Day, days of the lanternfly development which correspond to the developmental stages; day 0, hatching of the 1^st^ nymphal instar; day 74, appearance of the adults (based on dates reported in Dara et al. [8]).

We have also found a strong positive correlation between the labium length and the distance between tarsal claws of the forelegs during the lanternfly development (r = 0.63, n = 26, *P* < 0.001; S1 Fig); as well as very strong positive correlation for these traits when measurements were averaged across the developmental stages (r = 0.80, n = 5, *P* < 0.001). Growth of both the labium and tarsal tips is found to be hypoallometric in relation to the lanternfly growing stages (used as a proxy for the body size), with allometric coefficients of 0.29 and 0.34 for the labium length and distance between tarsal claw tips respectively (Fig 18).

## Discussion

From evolutionary and ecological perspectives, insect morphological adaptations for feeding and attachment on host plants can serve as constraints influencing insect diet breadth. Piercing-sucking mouthparts of the spotted lanternfly and adhesive tarsi facilitate effective host plant usage. Previous studies focused on the lanternfly behavior and pest management, and to the best of our knowledge only two studies explored mouthparts morphology and tarsal tips in adults using SEM [12,13]. Here we present the first detailed observations of developmental changes in the mouthparts and tarsal tips in the spotted lanternfly across 1^st^-4^th^ instar nymphs and adults. In this study we explored morphological variation in the labium, stylets, and tarsal tips using both SEM and morphometric analysis. We specifically focused on the morphological structures which presumably participate in the primary contact of the lanternfly with the host plant surface, and therefore, play essential role in host plant usage.

Our study revealed several interesting developmental patterns which potentially allow *L*. *delicatula* to better attach to a host plant and deeper penetrate to the host plant tissues at the late nymphal stages and adult stage: (a) the labium in adults consists of five segments whereas the nymphs have four labial segments; (b) the labium and stylet length, as well as the tarsal claw dispersal from the arolium, exponentially increase by 4^th^- instar nymph and adult stage; (c) mandibular stylets possess four indentations on the outer surface of the stylet apical part which become more evident in 4^th^-instar nymphs and adult; (d) longitudinal striations between indentations are present on mandibular stylets of 4^th^-instar nymphs and adults; (e) arolia surface becomes wrinkled in late instars and adults; and (f) changes in the angle of arolium growth follow the quadratic growth curve; the angle becomes obtuse in adults which potentially causes increase of the arolia basal width. Additionally, we have found six morphological types of sensilla which are present at the labial tip at each developmental stage of *L*. *delicatula*; which potentially indicates the lanternfly ability to effectively explore the host plant suitability at each developmental stage.

Overall, the substantial morphological and morphometric changes in mouthparts and tarsal tips were constantly observed for 4^th^ instars for each described characteristic. The application of these patterns to feeding activity and plant damage in late instars and adults can be explored further in future studies on the lanternfly host plant use.

### Labium and labial tip

Our study demonstrated that the labium length increases as the lanternfly grows, which is expected and might be associated with the increase in the stylet length, as well as the lanternfly body size [12]. We did not account for variation in the lanternfly body size in our study, but it is possible that body size changes are associated with the size of the head capsule and mouthparts [12]. Interestingly, the maximum width of the last labial segment differed little among stages, whereas the widest region had a different location in nymphs (at the labial tip) and adults (at the base of the last segment). Future studies might focus on how shape of the last segment and the size of the area of the labial tip affect host plant usage.

The length of the stylets and the last segment which we reported for the adults are comparable to the previous findings by Hao et al. [12], however the total labium length in the adult males which we reported is somewhat greater than that reported by Hao et al. (12957 ± 808 μm vs. 8132.02 ± 450.69 μm). It is possible that this difference in measurements might be due to either size differences between the introduced lanternfly population and its population in the native range (we collected adult males from the established North-American population in 4 years after the introduction of the spotted lanternfly in Pennsylvania); or due to different techniques and software used for measuring the labium length. Measuring the length of each labial segment was outside of the focus of our study, but future studies might consider measuring the length of each labial segment across all the developmental stages of the lanternfly to provide insights on population differences of the lanternfly in its native and introduced ranges.

We have also confirmed that 1^st^-4^th^ instar nymphs have 4-segmented labium, and the adults have 5-segmented labium, which was proposed in previous observations by Hao et al. [12]. The extra labial segment in adults (segment LS3) is located in the middle part of the labium which potentially may contribute to higher flexibility of the labium in adults and may facilitate deeper penetration to the host plant tissues (*J*. *Schultz*, *pers*. *comm*.). In this study, we did not focus on the morphology of segment LS3 and the comparison of its morphometrical characteristics with that in other labial segments. We observed that segment LS3 is a well-developed separate segment (Fig 2B and S4 Fig), but future studies might explore further whether the intersegmental membrane between segment LS3 and adjacent segments is visible and fully developed.

Labial sensilla are the first sensory organs which provides the lanternfly with its first contact with a host tree and facilitate host plant identification [12]. Brożek and Bourgoin [11] described nine types of sensilla on the labium of the spotted lanternfly. We observed six distinct morphological types of sensilla at each developmental stage. These morphological types were reported previously for adults only [12]. We have particularly focused on and described the bristle-like sensilla as their association with bark feeding was proposed in the previous studies [11]. It would be helpful for future studies to explore the functional role of each type of labial sensilla of *L*. *delicatula*. Some functional differences in the labial tip sensilla between adults and nymphs might be expected as the adults might need not only to select feeding sites but also suitable oviposition sites. Such developmental changes have been reported, for example, for cicada *Meimuna mongolica* [16]: the authors found that the number of sensilla and their sizes increased as the insect transitioned from 1^st^ nymphal stage to the adult stage. In cicadas, these developmental changes in sensilla might be associated with corresponding changes in host range as well as different microhabitats at each developmental stage [16]. For the lanternfly, it is also possible, from an evolutionary perspective, that extensive host selection and exploration in earlier instars are associated with larger sensory field than in the adults which are attached to one host plant for a long time.

### Stylets

In our study we focused on morphological variation in stylets among developmental stages, and particularly on the stylet tip morphology and stylet length. It has been demonstrated previously that the stylets in bark phloem feeders are especially adapted to pierce and penetrate thick plant tissues [17]. Our study revealed four indentations (oval prominences) on the apical surface of the mandibular stylets at each developmental stage of *L*. *delicatula*; these prominences, as well as longitudinal striations between them, become more evident in late nymphal instars and adults which can potentially be associated with penetration into thicker bark as the lanternfly grows. Particularly, Hao et al. [16] suggested that the number, size, and depth of such protrusions in hemipterans may reflect variation in host plant tissues and provide stronger anchoring as the insect body grows. Furthermore, previous studies indicate that the protrusions at the tip of the mandibular stylets help stabilize the maxillary stylets during probing [16].

The length of the stylets is another important factor influencing insect host selection [18]. Stylet lengths vary among hemipteran species, and might also reflect the type of tissues an insect attacks. In particular, insects that feed on stem phloem have the longest stylets [19]. Our study has also demonstrated that morphological changes in the stylet structures were accompanied by the changes in the stylet length. We showed that the stylet length increased as the lanternfly grows, and potentially its exposed part increased as well (based on data for 3^rd^ and 4^th^ instars only).

### Tarsal tip: tarsal claws and arolium

It has been shown previously that these parts of adult legs are about 5-fold and 6-fold as long as those of the 1^st^ instar stage [9]; no studies, however, have been done on morphological changes of tarsal tips across all the lanternfly developmental stages. Interestingly, our study revealed the increase in the arolia size (the width and the side length) only from 1^st^ to 4^th^ instar nymphs; we did not observe differences in the arolia size measurements between 4^th^ instar nymphs and adults. The latter might be associated with an expanded terminal sticky lip in adults which can be explored further in future studies. The increased arolium size along with the increased distance between tarsal claws in late instar nymphs and adults compared to that in early instar nymphs may reflect the pattern of host usage and correspond to the increased ability to grasp plant structures and stay longer on one host plant.

Following Frantsevich et al. [13] we also observed that the arolium surface was non-smooth and formed microscopical wrinkles. We recorded such contact splitting at each developmental stage, although the arolium surface in late instar nymphs and adults apparently possess more wrinkles than that in early instar nymphs. Future studies might focus on a detailed comparative analysis of such contact splitting among different developmental stages of the lanternfly. It has been demonstrated that contact splitting reduces the effect of substrate roughness and facilitate the effective attachment [20]. Such contact splitting potentially plays an important role in the lanternfly host plant usage as it facilitates the insect attachment and adaptability to microscopic irregularities of the plant surface [13]. Thus, insect attachment ability and host plant preferences may be affected by a plant surface profile [21]. Given the fact that bark surface has relatively rough surface compared to the surface of leaves and young branches, variation in the arolium surface may indicate the diet breadth at different developmental stages. Future studies might also focus on a comparative analysis of the tarsal tips in the lanternfly and related groups of Hemiptera in relation to their host plants.

### Potential implications for host plant usage

The results of morphological and morphometric analysis of this study are important for better understanding of host plant usage of the lanternfly during its development, and potentially for predicting the lanternfly host plants. Particularly, the stylet length and arolia adhesive properties may be critical for better attachment and utilization of the plant.

Previous studies on the whitefly demonstrated that the information of the stylet length may be helpful in investigating the mechanisms of stylet insertion [18]. It may be important for future studies to explore whether the lanternfly stylets penetrate plant tissue directly through epidermal cells or the stylets penetrate the plant tissue between epidermal cells. Studies on cicadas feeding have also shown that the stylet length is probably the determining factor for cicadas for choosing the feeding sites [22]. It has also been suggested that the late cicada instars can have longer stylets than the that in the adults; which allows nymphs to better anchor in the plant tissues during molting [22].

In general, the stylet size may correlate with the lanternfly body size [12]; however, various stylet length may also reflect variation in host plant tissues; particularly, it may indicate the fluid content of the tissue [16]. Also, the plant surface (e.g. wax) can affect the depth and success of stylet penetration [23]. Previous studies on aphids have also shown that the depth of stylet penetration may also correspond with insect starvation or wilting of the plant. For example, the frequency of probing may increase in starved insects [23].

As arolia adhesive properties decrease with the lanternfly age [13], it is also possible that 4^th^ instars, which have the stylet length and arolia size larger than that in the earlier nymphs, are the most active feeders and may cause more plant damage.

### Practical applications of the study

We specifically focused on growth and development of the labium, stylets, and tarsal tips. Applications of our work for future studies may include: (a) using a protocol for insect dissection, isolating arolia, and tissue preparation developed in this study for other investigations of the spotted lanternfly morphology; (b) estimating the intensity of plant damage based on the lanternfly stylet length and morphology; and (c) developing predictive models for the lanternfly host usage and dispersal based on plant surface profile.

Additionally, the described patterns in morphological variation which we observed for mouthparts and tarsal tips (specifically, patterns "b", "c", "d", and "e" described above) can be used for identification of the lanternfly nymphal instars. It is particularly critical for differentiation between 1^st^, 2^nd^, and 3^rd^ instars which have similar appearance while the size differences (between 1^st^ and 2^nd^, and between 2^nd^ and 3^rd^ instars) are not always obvious. For example, similarly to findings in Hao et al. [16] on cicadas morphology, the mouthpart length (such as the labium and the stylet length) in the lanternfly can indicate the nymphal stage. Also, we demonstrated that noticeable spread of the tarsal claws from the arolium was observed at 3^rd^ and 4^th^ nymphal stages only, which might help in differentiation between 2^nd^ and 3^rd^ instars.

## Supporting information

S1 Table The morphometric data of the labium and stylet fascicle of *Lycorma delicatula* at each developmental stage (original data). (CSV)

S2 Table The morphometric data of the tarsal claws and arolia of *Lycorma delicatula* at each developmental stage (original data). (CSV)

S1 Fig Correlation between the labium length and the distance between tarsal claws of the forelegs during the lanternfly development. (TIFF)

S2 Fig SEM of the arolium surface of the forelegs of adult female *Lycorma delicatula*. (A) Terminal sticky lip (Tsl). (B) Arolium dorsal surface. (C) Vertical slits of the terminal sticky lip (labeled following Frantsevich et al. [13]). (D) Surface of arolium wrinkles. Bars: (A) = 200 μm; (B) = 100 μm; (C) = 30 μm; (D) = 20 μm.
(TIF)

S3 Fig Schematic size and shape changes in the arolia of the forelegs of *Lycorma delicatula* across developmental stages. ᶿ, angle of the arolium growth.
(TIF)

S4 Fig SEM of the head and labial segments of adult female *Lycorma delicatula*. (A) Three last labial segments (Lb3, Lb4, and Lb5). (B) Extra labial segment (Lb3), which is not present in nymphs. Bars: (A) = 2 μm; (B) = 1 μm.
(TIF)

## References

[1] Bernays EA. Evolution of feeding behavior in insect herbivores. Bioscience. 1998; 48(1):35–44.

[2] Burkepile DE, Parker JD. Recent advances in plant-herbivore interactions. F1000Research. 2017;6. 10.12688/f1000research.10313.1 PMC5302155 28232868

[3] Barringer LE, Donovall LR, Spichiger SE, Lynch D, Henry D. The first new world record of *Lycorma delicatula* (Insecta: Hemiptera: Fulgoridae). Entomological news. 2015;125(1):20–4.

[4] Brożek J, Mróz E, Wylężek D, Depa Ł, Węgierek P. The structure of extremely long mouthparts in the aphid genus *Stomaphis* Walker (Hemiptera: Sternorrhyncha: Aphididae). Zoomorphology. 2015;134(3):431–45. 10.1007/s00435-015-0266-7 26346957 PMC4552766

[5] Barringer LE, Smyers E. Predation of the spotted lanternfly, *Lycorma delicatula* (White)(Hemiptera: Fulgoridae) by two native Hemiptera. Entomological News. 2016;126(1):71–4.

[6] Lee DH, Park YL, Leskey TC. A review of biology and management of *Lycorma delicatula* (Hemiptera: Fulgoridae), an emerging global invasive species. Journal of Asia-Pacific Entomology. 2019;22(2):589–596.

[7] Lee JE, Moon SR, Ahn HG, Cho SR, Yang JO, Yoon CM, Kim GH. Feeding behavior of *Lycorma delicatula* (Hemiptera: Fulgoridae) and response on feeding stimulants of some plants. Korean journal of applied entomology. 2009;48(4):467–77.

[8] Dara SK, Barringer L, Arthurs SP. *Lycorma delicatula* (Hemiptera: Fulgoridae): a new invasive pest in the United States. Journal of Integrated Pest Management. 2015;6(1):20.

[9] Kim JG, Lee EH, Seo YM, Kim NY. Cyclic behavior of *Lycorma delicatula* (Insecta: Hemiptera: Fulgoridae) on host plants. Journal of Insect Behavior. 2011;24(6):423.

[10] Backus EA. Anatomical and sensory mechanisms of leafhopper and planthopper feeding behavior. The leafhoppers and planthoppers. 1985:163–94.

[11] Brożek J, Bourgoin T. Morphology and distribution of the external labial sensilla in Fulgoromorpha (Insecta: Hemiptera). Zoomorphology. 2013;132(1):33–65. 10.1007/s00435-012-0174-z 23420415 PMC3570763

[12] Hao Y, Dietrich CH, Dai W. Structure and sensilla of the mouthparts of the spotted lanternfly *Lycorma delicatula* (Hemiptera: Fulgoromorpha: Fulgoridae), a polyphagous invasive planthopper. PloS one. 2016;11(6):e0156640 10.1371/journal.pone.0156640 27253390 PMC4890751

[13] Frantsevich L, Ji A, Dai Z, Wang J, Frantsevich L, Gorb SN. Adhesive properties of the arolium of a lantern-fly, *Lycorma delicatula* (Auchenorrhyncha, Fulgoridae). Journal of insect physiology. 2008;54(5):818–27. 10.1016/j.jinsphys.2008.03.005 18479702

[14] R Core Team. R: A language and environment for statistical computing; R Foundation for Statistical Computing: Vienna, Austria, 2014; Available online: http://www.R-project.org/.

[15] Laforsch C, Tollrian R. A new preparation technique of daphnids for scanning electron microscopy using hexamethyldisilazane. Archiv für Hydrobiologie. 2000 11 24:587–96.

[16] Hao Y, Dietrich CH, Dai W. Development of mouthparts in the cicada *Meimuna mongolica* (Distant): successive morphological patterning and sensilla differentiation from nymph to adult. Scientific reports. 2016;6:38151 10.1038/srep38151 27901084 PMC5128874

[17] Ge F, Dietrich C, Dai W. Mouthpart structure in the woolly apple aphid *Eriosoma lanigerum* (Hausmann)(Hemiptera: Aphidoidea: Pemphigidae). Arthropod structure & development. 2016;45(3):230–41. 26806553 10.1016/j.asd.2016.01.005

[18] Freeman TP, Buckner JS, Nelson DR, Chu CC, Henneberry TJ. Stylet penetration by *Bemisia argentifolii* (Homoptera: Aleyrodidae) into host leaf tissue. Annals of the Entomological Society of America. 2001;94(5):761–8.

[19] Brożek J, Mróz E, Wylężek D, Depa Ł, Węgierek P. The structure of extremely long mouthparts in the aphid genus *Stomaphis* Walker (Hemiptera: Sternorrhyncha: Aphididae). Zoomorphology. 2015;134(3):431–45. 10.1007/s00435-015-0266-7 26346957 PMC4552766

[20] Kim JK, Varenberg M. Contact splitting in dry adhesion and friction: reducing the influence of roughness. Beilstein Journal of Nanotechnology. 2019;10(1):1–8. 30680274 10.3762/bjnano.10.1 PMC6334799

[21] Al Bitar L, Voigt D, Zebitz CP, Gorb SN. Attachment ability of the codling moth *Cydia pomonella* L. to rough substrates. Journal of Insect Physiology. 2010;56(12):1966–72. 10.1016/j.jinsphys.2010.08.021 20816976

[22] Leopold RA, Freeman TP, Buckner JS, Nelson DR. Mouthpart morphology and stylet penetration of host plants by the glassy-winged sharpshooter, *Homalodisca coagulata*, (Homoptera: Cicadellidae). Arthropod structure & development. 2003;32(2–3):189–99. 18089004 10.1016/S1467-8039(03)00047-1

[23] Pollard DG. Plant penetration by feeding aphids (Hemiptera, Aphidoidea): a review. Bulletin of Entomological Research. 1973;62(4):631–714.

