# Supplementary figures and images for: External morphology and developmental changes of tarsal tips and mouthparts of the invasive spotted lanternfly, Lycorma delicatula (Hemiptera: Fulgoridae)
Source: PLoS One. 2019 Dec 26;14(12):e0226995. doi: 10.1371/journal.pone.0226995 (PMC6932783; doi:10.1371/journal.pone.0226995)

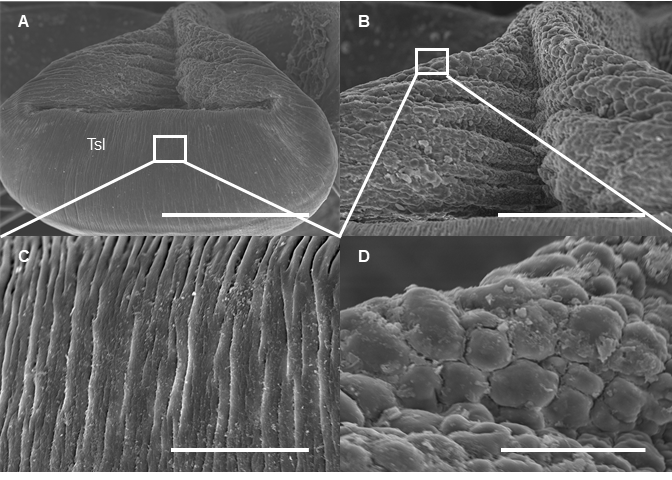

Supplement: S2 Fig — (A) Terminal sticky lip (Tsl). (B) Arolium dorsal surface. (C) Vertical slits of the terminal sticky lip (labeled following Frantsevich et al. [13]). (D) Surface of arolium wrinkles. Bars: (A) = 200 μm; (B) = 100 μm; (C) = 30 μm; (D) = 20 μm. (TIF) [file pone.0226995.s004.tif]

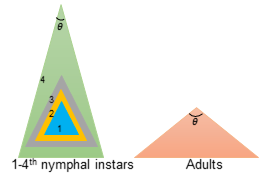

Supplement: S3 Fig — ᶿ, angle of the arolium growth. (TIF) [file pone.0226995.s005.tif]

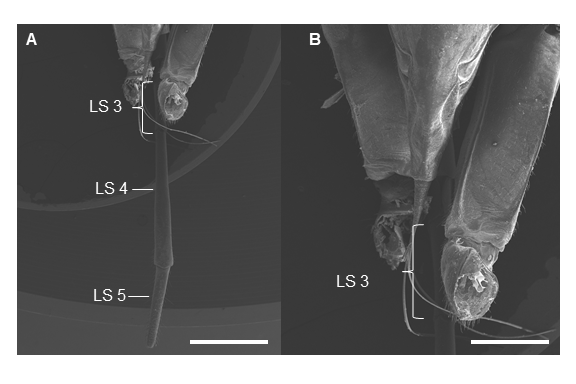

Supplement: S4 Fig — (A) Three last labial segments (Lb3, Lb4, and Lb5). (B) Extra labial segment (Lb3), which is not present in nymphs. Bars: (A) = 2 μm; (B) = 1 μm. (TIF) [file pone.0226995.s006.tif]
